# Resource Availability Modulates the Cooperative and Competitive Nature of a Microbial Cross-Feeding Mutualism

**DOI:** 10.1371/journal.pbio.1002540

**Published:** 2016-08-24

**Authors:** Tim A. Hoek, Kevin Axelrod, Tommaso Biancalani, Eugene A. Yurtsev, Jinghui Liu, Jeff Gore

**Affiliations:** 1 Hubrecht Institute, The Royal Netherlands Academy of Arts and Sciences (KNAW) and University Medical Center Utrecht, Utrecht, the Netherlands; 2 Biophysics PhD Program, Harvard University, Cambridge, Massachusetts, United States of America; 3 Physics of Living Systems, Department of Physics, Massachusetts Institute of Technology, Cambridge, Massachusetts, United States of America; Hebrew University, ISRAEL

## Abstract

Mutualisms between species play an important role in ecosystem function and stability. However, in some environments, the competitive aspects of an interaction may dominate the mutualistic aspects. Although these transitions could have far-reaching implications, it has been difficult to study the causes and consequences of this mutualistic–competitive transition in experimentally tractable systems. Here, we study a microbial cross-feeding mutualism in which each yeast strain supplies an essential amino acid for its partner strain. We find that, depending upon the amount of freely available amino acid in the environment, this pair of strains can exhibit an obligatory mutualism, facultative mutualism, competition, parasitism, competitive exclusion, or failed mutualism leading to extinction of the population. A simple model capturing the essential features of this interaction explains how resource availability modulates the interaction and predicts that changes in the dynamics of the mutualism in deteriorating environments can provide advance warning that collapse of the mutualism is imminent. We confirm this prediction experimentally by showing that, in the high nutrient competitive regime, the strains rapidly reach a common carrying capacity before slowly reaching the equilibrium ratio between the strains. However, in the low nutrient regime, before collapse of the obligate mutualism, we find that the ratio rapidly reaches its equilibrium and it is the total abundance that is slow to reach equilibrium. Our results provide a general framework for how mutualisms may transition between qualitatively different regimes of interaction in response to changes in nutrient availability in the environment.

## Abstract 2

A combination of computational modeling and experiments reveals the striking effects of changing resource availability on the population dynamics observed between two cross-feeding yeast strains.

## Author Summary

Species often engage in mutualistic interactions that are beneficial for both partners. However, there is also a cost associated with cooperation, for example, in the form of energy required to make nutrients for a partner. When environments change, the costs and benefits of cooperating can change as well, and this can cause the mutualistic interaction to break down into other interaction types, such as parasitism. In this study, we varied nutrient availability to examine how changing environments can affect the interaction between two cross-feeding yeast strains. Lower nutrient concentrations made each strain more dependent on the nutrients provided by its partner strain and thus favored cooperation. Using both experiments and mathematic models, we found that in different environments, these yeast strains can interact in at least seven different qualitatively different ways, including obligate mutualism, facultative mutualism, parasitism, and competition. We also found that the dynamics of how the two strains influence each other change drastically in different nutrient concentrations. Examining the population dynamics could therefore potentially be used to predict the stability or collapse of a community.

## Introduction

Species in a community interact in a bewildering variety of ways, from parasitic to competitive to mutualistic. Mutualisms, in which two species engage in reciprocal cooperative behavior that benefits both partners, are thought to be particularly important for the stability of ecosystems [1,2], although recent work questioned this role of cooperation in ecosystem stability [3]. Mutualisms in nature are common and diverse, including the pollination of crops and other plants by bees [4], the cross-protection between clown-fish and anemone [5], and the symbiosis between tubeworms and bacteria [6]. In the case of the tubeworm, the interaction is completely obligatory because it has no digestive system and acquisition of energy depends completely on bacterial symbionts. The mutualism between most plants and their pollinators, however, is typically facultative, as most plants have multiple pollinators and most pollinators feed from multiple plant species.

Within the microbial realm, mutualisms can be due to cross-protection [7] or due to cross-feeding, in which each species supplies their partner with nutrients. Cross-feeding interactions can be present within a species [8], between pairs of species [9–11], or could represent a complicated network of dependencies [12] and possibly play a major role in driving the diversity of microbial communities in environments such as the soil [13,14]. In addition, cross-feeding could play an important role in determining the species composition and community-level functioning within the human gut microbiome [15]. Laboratory experiments are ideal for studying cross-feeding mutualisms, as they enable fine-grained control of microbial populations and the resources available in the environment. This provides the potential to integrate experiments and models in ways not possible in the field. For example, laboratory experiments have been used to show that cross-feeding can have a stabilizing effect on the relative abundance of two microbial species [9], which can protect against invasion by cheater strains [16].

Although species in a mutualism generally benefit from interacting with each other, these benefits might decrease in different environments. A major focus of recent research on mutualisms has attempted to elucidate the conditions in which a mutualism can break down or switch to parasitism [17,18]. For example, the cross-protection mutualism between ants and the plants that house them can break down when grazing pressure on the plant is reduced [19], and mycorrhizal mutualisms can become parasitic in the absence of abiotic stresses [20]. Theoretical work predicts that certain mutualisms can become competitive in high nutrient conditions [21]. Moreover, a global analysis of plant interactions concluded that interactions were often facilitative in the challenging environments present at high elevation, whereas the interactions became increasingly competitive in the more benign environments at low altitudes [22]. More generally, the mutualism–parasitism continuum hypothesis posits that a number of environments may cause a mutualism to degrade into a parasitic interaction [23]. Conversely, exposure to certain challenging environments that favor cooperation can stimulate establishment of novel mutualistic interactions [24,25], and theoretical work predicted that almost any pair of species in a microbial ecosystem could establish cooperative interactions when grown in the right nutrient conditions [26]. Resource availability can also alter features other than the growth rate of cooperative strains. For example, resource availability can affect the spatial structure of cooperative species in a biofilm [27,28], as well as the degree of intermixing of cooperative strains during a range expansion [10,29]. Although multiple studies have observed a shift in interaction because of varying environmental conditions, a detailed understanding of these changes is missing. It is currently unknown what the possible interaction shifts are and how the population dynamics of a mutualism are affected by these shifts.

In our work, we use a synthetic cross-feeding yeast system in which we can modulate the relative strength of the mutualistic and competitive aspects of the interaction by supplementing the media with the amino acids that the strains cross-feed. By changing these two nutrient concentrations, we are able to switch between a surprisingly large number of different interaction types, including obligatory and facultative mutualism, competition, parasitism, competitive exclusion, and extinction of the population. Each of these regimes shows qualitatively different dynamics, which we can understand using a simple model. Our experiments shed light on the important question of how resource availability can modulate the types of interaction between species in a mutualism.

## Results

### Cross-Feeding Results in a Stable Mutualism

**Fig 1:**
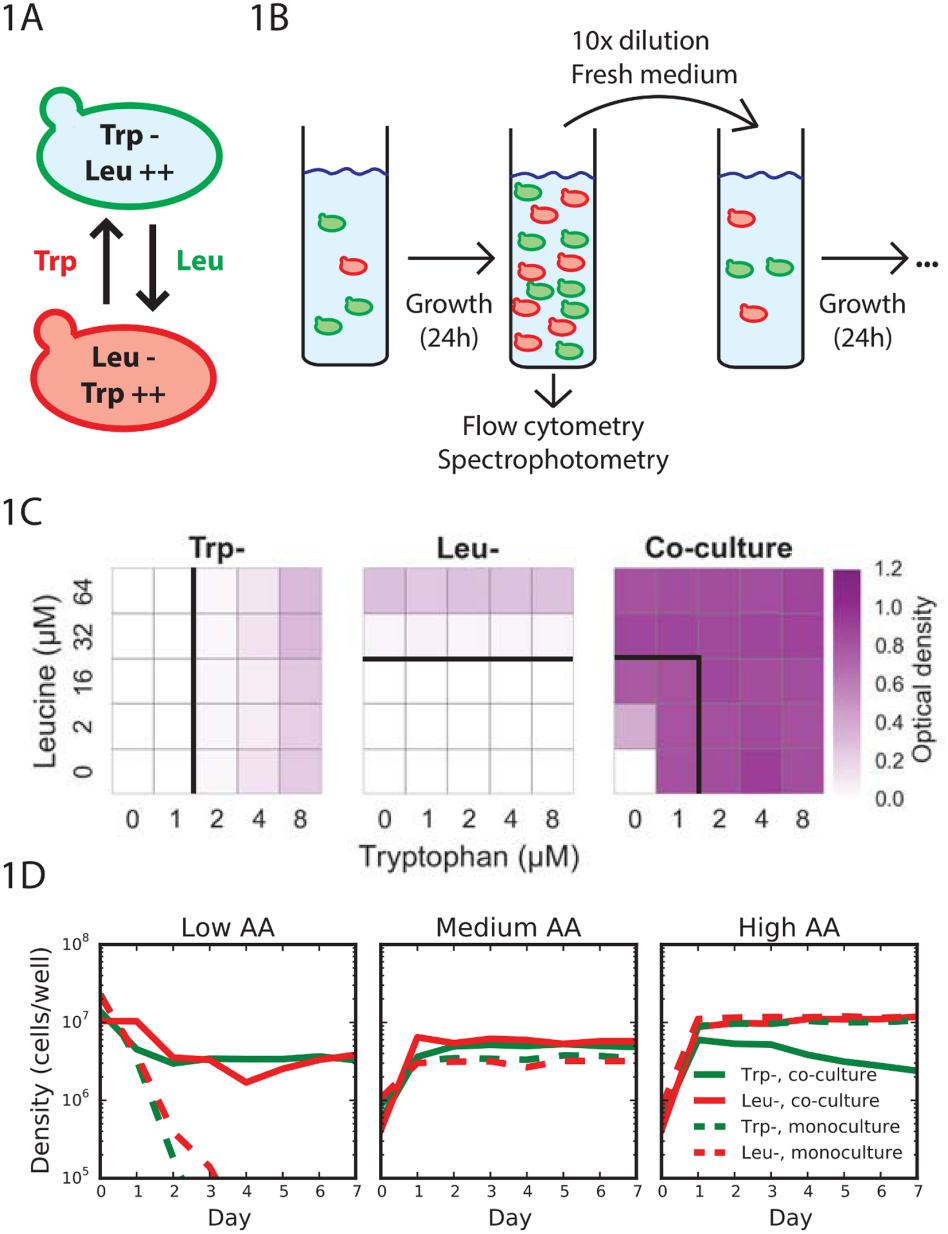
Two auxotrophic yeast strains can form a stable cross-feeding mutualism in a range of nutrient concentrations. (A) The YFP-tagged strain is unable to produce the amino acid tryptophan but overproduces the amino acid leucine, whereas the RFP-tagged strain is unable to produce leucine but overproduces tryptophan. (B) The mutualism is probed by co-culturing the two auxotrophic yeast strains in batch culture with 10x dilution daily. Flow cytometry and spectrophotometry report on the relative fraction and total abundance of the two yeast strains at the end of each day of growth. (C) Optical density after 8 d of daily dilution and growth. The co-culture is able to survive in low amino acid concentrations where the monocultures cannot survive (solid lines indicate the concentrations below which each auxotrophic strain goes extinct). (D) Abundance of the co-culture (solid line) and monocultures (dashed line) for the Trp^-^ (green) and Leu^-^ (red) strains. In low amino acid concentrations (1 and 8 μM), the strains form an obligate mutualism; in medium amino acid concentrations (8 and 64 μM), the strains form a facultative mutualism; and in high amino acid concentrations (32 and 256 μM), the strains form an amensalism, as the Leu^-^ strain is relatively unaffected and the Trp^-^ strain is harmed by the interaction.

As a model system for mutualistic interactions, we used two non-mating *Saccharomyces cerevisiae* budding yeast strains that have been engineered to be deficient in the biosynthesis of an essential amino acid and also overproduce the amino acid required by its partner (Fig 1A) [10]. The red fluorescent protein (RFP)-tagged leucine auxotrophic strain (Leu^-^) overproduces tryptophan, whereas the yellow fluorescent protein (YFP)-tagged tryptophan auxotroph strain (Trp^-^) overproduces leucine. These strains have previously been demonstrated to form a cross-feeding mutualism when grown on solid agar, with each strain leaking out the amino acid needed by its partner [10].

To determine if we could establish a stable mutualism between these strains in well-mixed liquid batch culture, we inoculated monocultures and co-cultures at a range of leucine and tryptophan concentrations (Fig 1B and 1C). Co-cultures were started with equal amounts of each strain at the same total density as monocultures. Each day we diluted by a factor of ten into fresh media containing the same defined concentrations of leucine and tryptophan (Fig 1B). For a culture to survive, the growth of a population during the day should be at least as large as the decrease caused by dilution, and a population thus needs to divide at least log_2_(10) = 3.3 times each day. In monoculture, Trp^-^ cells required at least 2 μM tryptophan to avoid going extinct due to dilution, whereas Leu^-^ cells required a minimum of 32 μM leucine. In contrast, co-cultures could survive on concentrations of leucine and tryptophan where the monocultures would each go extinct. Co-cultures survived eight of these growth-dilution cycles, indicating a stable mutualism. Even in concentrations where monocultures survived, we found that co-culture density was often much higher than the sum of monoculture densities (Fig 1C), suggesting that in this regime the strains were interacting in a facultative mutualism.

### Amino Acid Supplementation Makes the Interaction More Competitive

Understanding the relative benefits that each partner in the mutualism does or does not receive requires that we also determine the population abundance of each strain at different amino acid concentrations. We therefore co-cultured the strains and measured the population composition by flow cytometry at the end of each day. We tried to make both strains receive equal benefits from the amino acids being supplemented by adding leucine and tryptophan in a ratio of 8 to 1, which is approximately the intracellular ratio of these amino acids [30]. We found that at low amino acid concentrations (1 μM tryptophan, 8 μM leucine; 1 and 8 μM), the strains indeed form an obligate mutualism with an apparently stable coexistence, because relative abundance changes little over time (Fig 1D). At medium amino acid concentrations (8 and 64 μM), the strains form a facultative mutualism, with both strains benefiting from the presence of the other strain, yet also surviving when grown in monoculture. At high amino acid concentrations (32 and 256 μM), we observed coexistence of the two strains, but with the Trp^-^ strain at an equilibrium abundance below what it would have reached in a monoculture. At this high amino acid concentration, we therefore found that the strains are forming an amensalism, in which the Leu^-^ strain is relatively unaffected by the interaction but the Trp^-^ strain performs worse in co-culture than in monoculture. This demonstrates that a simple microbial cross-feeding mutualism can transition into a qualitatively different interaction by a simple change in environmental conditions.

Throughout our study, we compare the final population size of each strain in monoculture and co-culture to assess whether each strain is benefitted, harmed, or unaffected by the presence of its partner in each environmental condition. Once populations have reached an equilibrium size, all populations have the same mean growth rate over the course of the day, because reaching the same population size after a cycle of dilution and growth requires that each cell type undergo log_2_(10) = 3.3 divisions over the course of the day. The division rate of a population is therefore not an appropriate measure of fitness or benefit/harm from a partner, as the division rate at equilibrium is always the same given the constant dilution rate present within the experiment. We also note that throughout each daily cycle of growth, the strains alter their habitat by consuming and producing amino acids. Therefore, the label for the different environments (e.g., 2 μM tryptophan and 16 μM leucine) corresponds to the amino acid concentration of the media that we use to initialize growth at the beginning of each day.

### Phenomenological Model Predicts Transitions between Qualitatively Different Regimes of Interaction

To gain insight into the transition between the different regimes of interaction in our cross-feeding strains, we implemented a simple phenomenological model designed to capture the essential elements of the interactions between the strains. We assumed that the two strains Trp^-^ (X) and Leu^-^ (Y) have a per capita growth rate that is modulated by the mutualistic partner as well as the supplemented amino acids:

$$\frac{dX}{dt}=r_{x}X\;\left(\frac{Y+a}{Y+a+\kappa }\right)\left(\;1-X-Y\right)-\delta X$$ (1)

$$\frac{dY}{dt}=r_{y}Y\;\left(\frac{\beta X+a}{\beta X+a+\kappa }\right)\left(\;1-X-Y\right)-\delta Y$$ (2)

Here *r*_*x*_ and *r*_*y*_ are the growth rates, *a* is the amount of supplemented amino acids, δ is the death rate imposed by dilution, κ is an effective Monod constant, and β quantifies the asymmetry of benefit that each strain receives from its partner. The growth rate of each strain increases with the abundance of the mutualist partner and the needed amino acid, but this benefit saturates via a Michaelis-Menten/Monod form as a function of both the concentration of the partner and the supplemented amino acid. This particular form for the interaction arises from a resource-explicit model in which the amino acid dilution/degradation is larger than consumption, but the qualitative predictions of the model are robust to this assumption (S1 Information). We assume that the supplemented amino acids are always added at a fixed ratio, so we use a single variable “*a*” to capture the amount of supplemented amino acids (despite the fact that the two strains are actually consuming different amino acids). Because the 1-to-8 ratio of tryptophan to leucine should give about equal “relative” amounts of amino acids, we used the same scaling constant (κ = 0.12) for both equations. The two strains are also assumed to use other resources in the environment and hence saturate at a total population size, which is normalized to 1. Additionally, we recapitulated our daily dilutions by introducing a fixed death rate, δ = 0.5 (although our experiments are done in batch culture, for simplicity we model our mutualism in continuous culture). We incorporated only two aspects of the asymmetry between our two strains. First, based on competition experiments in saturating amino acid concentrations (200 and 1,600 μM), we calculated that Leu^-^ has a fitness disadvantage of ~7.5% in optimal conditions (S1 Fig), so we set the normalized growth rates to be r_x_ = 1 and r_y_ = 0.925. Second, we assume that the Trp^-^ strain contributes more nutrients to the mutualism than the Leu^-^ strain (β = 2) because the Leu^-^ strain dominated co-cultures at non-saturating amino acid concentrations (Fig 1D, also see below).

**Fig 2:**
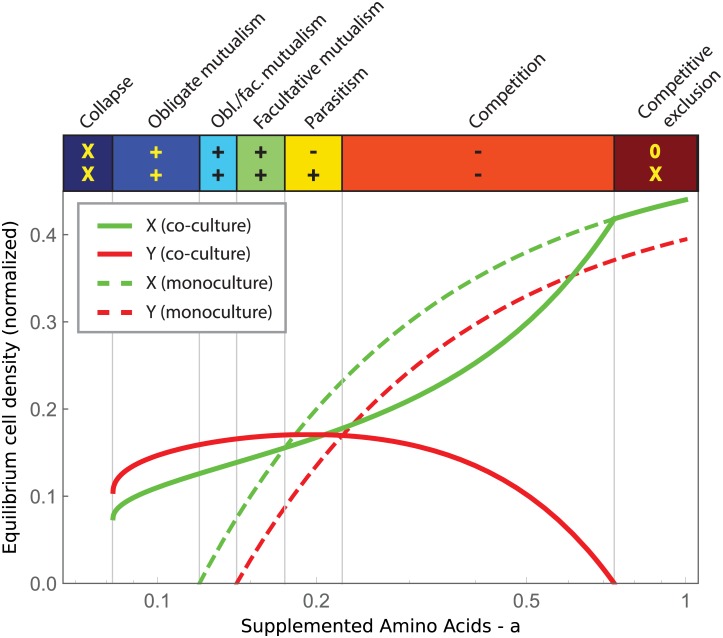
A simple phenomenological model predicts that a cross-feeding mutualism can shift between many qualitative outcomes. Plot shows equilibrium density of co-cultures (solid lines) or monocultures (dashed lines) as a function of amino acids. The colorbar above the plot shows the qualitative regimes of interaction and indicates the effect of growth in co-culture for both strains in comparison with their growth in monoculture. Effects of growing in co-culture can be beneficial (+), harmful (-), neutral (0), or leading to extinction (X).

This simple phenomenological model was able to explain the qualitative regimes of interactions that we observed previously (Fig 1D) and suggested that simply by varying the amino acid concentrations we may be able to observe an even larger number of qualitative outcomes between our two strains (Figs 2 and S7). Increasing amino acid concentrations from the region of obligatory mutualism (Fig 2, blue), the model predicts that the interaction should become a facultative mutualism (green) followed by a parasitism (yellow), with the Leu^-^ benefiting from the interaction and the Trp^-^ being harmed. The model then predicts that the amensalism previously observed in Fig 1D corresponds to the boundary of the parasitism region and a competition region (orange), in which the strains coexist but at an equilibrium density below what they would reach in monoculture. This outcome is achieved despite the fact that the force leading to coexistence of the strains is still the sharing of amino acids. Since these strains have complete niche overlap, coexistence is not possible without a stabilizing influence, which is provided by amino acid transfer [31]. At even higher amino acid concentrations the model predicts that the strain with a higher maximal growth rate (Trp^-^) should outcompete the slower dividing strain, because in this regime, amino acids are no longer limiting (Competitive Exclusion, red). The model also predicts that due to the asymmetry in the strains, there will be a small region where the interaction is a facultative mutualism for one strain yet an obligatory mutualism for the other strain (cyan). Finally, the model predicts that in the absence of supplemented amino acids, the mutualism will fail and both strains will go extinct (dark blue). These results are not the result of a particular parameter setting, as the model predicts a shift through the same qualitative regimes over a large range of values for the death rate δ (S8 Fig). This model, although exceedingly simple, therefore predicts the existence of a surprisingly wide range of different qualitative outcomes within a mutualist pair.

### Experimental Confirmation That These Cross-Feeding Strains Can Transition between a Wide Range of Different Qualitative Types of Interactions

**Fig 3:**
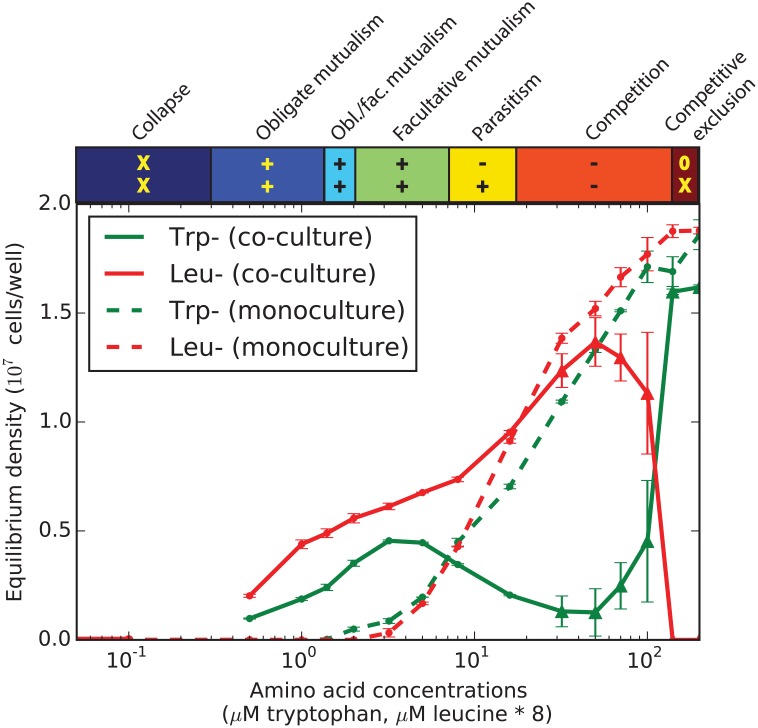
Our experimental cross-feeding mutualism shifts between the predicted eight different qualitative outcomes. Co-cultures were grown at amino acid concentrations ranging from 0 to 200 μM Tryptophan and 0 to 1,600 μM Leucine. Leucine concentrations were 8 times higher than tryptophan concentrations in all conditions. Cultures were started at 6 different fractions and run for 7 d with a 10x dilution each day. In cultures that reached equilibrium (small dots), data shows mean density (+- standard error of the mean [s.e.m]), whereas in cultures that did not reach equilibrium (larger triangles), mean equilibrium density is estimated based on growth (+- s.e.m. S2 and S3 Figs, S1 Information). Colorbar above the plot shows the qualitative regimes of interaction as in Fig 2.

To test these model predictions of many different interaction regimes, we experimentally measured the equilibrium abundances at a wide range of amino acid concentrations (Figs 3 and S2). As predicted by the model, we found that varying the amino acid concentration caused the mutualist pair to switch between seven different qualitative regimes, with the ordering of these regimes as predicted by the model. From low to high amino acid concentrations, we observed collapse of the mutualism, obligatory mutualism, obligatory/facultative mutualism (different for the two strains), facultative mutualism, parasitism, competition, and competitive exclusion. Note that there are slight differences between the model and experiment in the behavior of the monocultures, as the Leu^-^ strain is more abundant than the Trp^-^ strain at high amino acid concentrations in our experiment. Nevertheless, it is remarkable that such a simple model provides such effective guidance in the outcomes that we observe in our experimental microbial cross-feeding system.

### Characteristic Behaviors before Population Collapse

In both the model (Fig 2) and in the experimental system (Fig 3), the two strains coexist for intermediate values of supplemented amino acids, but one or both strains go extinct if the amount of supplemented amino acids is either too small or too large. This means that if the environment were to deteriorate (for example, by decreasing nutrient availability), the system would go through a series of changes in the type of interaction (e.g., parasitism, facultative mutualism) before becoming an obligatory mutualism and finally going extinct due to the environmental deterioration. Similarly, a rich environment would render the mutualism ineffective, so that the strain with lower fitness would eventually be outcompeted by the other. In principle, knowing the interaction type would indicate whether the system is approaching extinction, although this information requires knowledge of the equilibrium densities for both monocultures and co-cultures, which may not be easily available for many natural systems.

**Fig 4:**
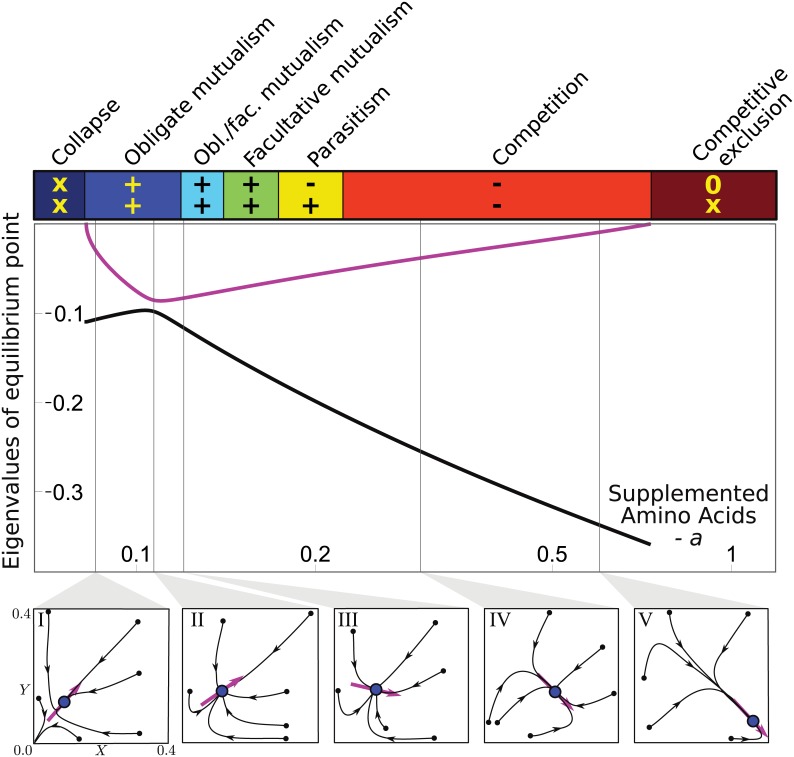
Eigenvector orientation predicts characteristic behaviors prior to population collapse. Main plot shows the two eigenvalues as a function of supplemented amino acids (black and magenta solid lines). Population collapse occurs when the slower eigenvalue (magenta solid line) reaches zero. Insets I–V show simulated population trajectories (solid, arrowed black lines) for different amino acid concentrations and starting from different initial population densities (small black dots). The eigenvector (magenta arrow) of the stable equilibrium point (blue dot) corresponds to the slower eigenvalue. Insets show that trajectories align to the eigenvector when the system is close to collapse. This indicates that the ratio of the densities of each strain within the population (i.e., *f* = *X*/*Y*) relaxes faster than the total population size (i.e., *n* = *X*+*Y*) when the system is close to extinction (inset I), whereas the opposite scenario occurs before the competitive exclusion regime (inset V).

An alternative way to detect an imminent population collapse consists of looking at early-warning signals, which are characteristic features exhibited by biological populations prior to an abrupt change of state [32]. To this end, we have analyzed the model behavior near the two onsets of extinction, namely in the obligatory mutualism and competition regimes. The equilibrium densities of the two strains in co-culture are given by the single non-zero equilibrium point of Eqs 1 and 2 (Fig 2). This equilibrium is stable, meaning that the system recovers from small perturbations in the way described by its eigenvalues and eigenvectors (Fig 4). The two eigenvalues, both negative, indicate how rapidly the equilibrium point is approached by the population trajectories along the directions given by the corresponding eigenvectors. A large negative eigenvalue indicates a rapid convergence (i.e., solid black line), whereas a small negative value indicates a slow convergence (i.e., solid magenta line). At nutrient concentrations near the onset of extinction, both in the obligatory mutualism or competition regimes, there is a separation of time scales: the slow eigenvalue goes to zero, indicating that the system takes a long time to reach the equilibrium point (blue dot in insets) along the slow eigenvector (magenta arrow in insets). Simulations of the model confirm that near the onsets of extinction, the trajectories align parallel to the slow eigenvector before reaching the equilibrium point (insets I, IV, and V)—a phenomenon that does not occur when the eigenvalues assume similar values (insets II and III). Finally, the orientation of the slow eigenvector indicates which quantity is slowly relaxing: close to collapse of the mutualism (inset I), the ratio of the densities of each strain within the population (i.e., *f* = *X*/*Y*) relaxes faster than the total population size (i.e., *n* = *X*+*Y*); in contrast, before competitive exclusion occurs (inset V), the population quickly converges to a fixed *n*, while slowly equilibrating *f* to the amount determined by the equilibrium point.

In summary, our model predicts that the approach to equilibrium is very different when the cross-feeding strains interact in an obligatory mutualism as compared to when they interact competitively (Fig 4, see Materials and Methods section). Competitively interacting strains rapidly reach carrying capacity, and only later does the ratio of the strains reach equilibrium (Fig 4, inset V). In contrast, in the obligatory mutualism regime close to collapse, it is the ratio that first reaches equilibrium, and the total population size is the variable that is slow to reach equilibrium (Fig 4, inset I). In between these two interaction regimes there is no separation of timescales, and the approach to equilibrium is predicted to be approximately uniform from all directions (Fig 4, insets II and III). These changes in dynamics are expected very generally due to critical slowing down, in which the slow relaxation mode is associated with the direction of the eigenvector as the eigenvalue goes to zero (Fig 4). The model therefore predicts that simply measuring the dynamics of the partner strains allows for an estimate of the kind of interaction and, hence, how close the population is to collapse.

**Fig 5:**
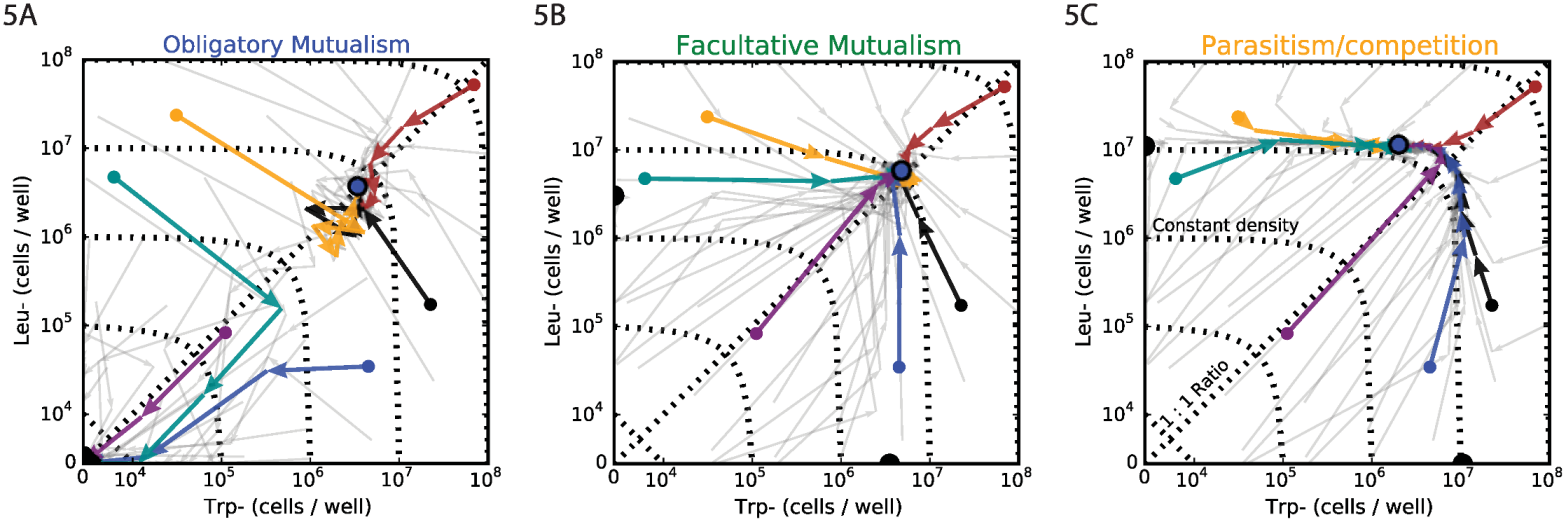
Relaxation dynamics is observed before population collapse. In each figure, we started co-cultures at 48 different population sizes (*n* = *X*+*Y*) and relative abundances (*f* = *X*/*Y*). Six co-cultures per figure are highlighted (colored arrows), with day 0 density represented by a colored dot and each arrow signifying the change over a single day. Black dots on the axes indicate monoculture equilibria, whereas the blue dot shows the co-culture equilibrium. (A) In obligatory mutualistic conditions, close to extinction, populations approach equilibrium from the direction corresponding to constant ratio, *f*. (B) In medium amino acid concentrations, there is no privileged direction for approaching equilibrium. (C) At high concentrations, when the two strains compete against each other, the population approaches equilibrium from the constant *n* manifold. Axes are linear at densities of 0 to 10^4^ cells/well and logarithmic for higher densities.

In order to test these model predictions, we measured the dynamics of co-cultures initialized at a wide range of population sizes *n* and starting ratios *f*, spanning four and eight orders of magnitude, respectively (Fig 5). In accordance with the predictions of the model, in high amino acid concentrations (32 μM tryptophan and 256 μM leucine), we observed rapid convergence of *n*, whereas *f* did not equilibrate even after five days (Fig 5C). In contrast, in low amino acid conditions (1 μM tryptophan and 8 μM leucine, Fig 5A), the interaction is an obligatory mutualism and the cross-feeding interaction resulted in a strong stabilizing effect on the relative abundances [9], with the populations rapidly reaching a 1-to-1 ratio (i.e., *f* = 1). As *f* equilibrated, the fate of the populations depended on the population size *n*: those that started at sufficiently high abundance slowly increased their total population size to the equilibrium point value, whereas populations that started too small or imbalanced were fated to extinction (*n* = 0). We were therefore able to experimentally observe the two different separations of timescale predicted by the model in the two different extreme regimes of interaction. Finally, we found that at intermediate amino acid concentrations (8 μM tryptophan and 64 μM leucine), there was a balance between the two relaxation timescales, thus causing the trajectories to converge to equilibrium from all directions (Fig 5B) as predicted by the model (Fig 4 insets II and III). Therefore, the relaxation dynamics of the cross-feeding partners provide an early-warning indicator of population collapse.

## Discussion

We have established an experimental system that captures a multitude of interactions by simply varying the amount of nutrients freely available to two partners in a cross-feeding mutualism. Although it is tempting to conclude that this cross-feeding interaction should be an obligatory mutualism, we demonstrate experimentally that the interaction varies greatly with the environment. Depending upon the environment, we found that our cross-feeding strains could interact as an obligatory or facultative mutualism, parasitism, amensalism, or competition. A simple phenomenological model explained this range of outcomes, which we view as a significant success given that many models of mutualisms have difficulty shifting between such qualitatively different outcomes; indeed, the Lotka–Volterra model of interspecies interactions fails to even describe an obligatory mutualism without leading to ever-expanding populations [33]. Moreover, the model predicts different relaxation time scales on the brink of collapse that have been confirmed in our experimental system.

Our experiments and modeling suggest that the interaction becomes increasingly cooperative as the environmental quality deteriorates via decreasing nutrient availability. This observation is consistent with work done on a range of other mutualisms and interspecies interactions [16,19,20,22,26]. However, our results show a much greater range of possible interactions than demonstrated previously and strengthen the idea of interactions between species being contingent upon the environmental conditions rather than being fixed.

We also found that the population dynamics change drastically with changing nutrient availability. Low nutrient concentrations have a strong stabilizing effect on relative abundance, whereas high nutrient concentrations stabilize total population size. These dynamics provide a possible way to estimate the interaction and stability of a potential mutualism without having data regarding the viability of each species on its own. The strong stabilizing effect on either total population size or relative abundance suggests that variation will be predominantly on the variable that is not strongly stabilized. In particular, at low nutrient availability, fluctuations may lie primarily along the total population size, whereas in high nutrient availability, the fluctuations in relative abundance may be larger. These differences could provide a more accessible way of studying the stability of species with positive interactions, as it requires only studying the fluctuations of the populations around their equilibrium. Moreover, an experimentally tractable cross-feeding system such as ours could be used to explore counterintuitive effects predicted to occur as a result of noise, such as enhanced sensitivity to environmental fluctuations [34] and noise-induced oscillations [35].

In our study, we focused on the ecological dynamics of mutualisms (changes in the number of individuals in a population) rather than evolutionary dynamics (changes in genetic structure). Rather than asking questions about how two strains would evolve cross-feeding, we simply assumed a priori that such an interaction had arisen evolutionarily. Given such an interaction as a starting point, we sought to understand the environmental conditions under which the mutualism would transition into competition. It would be fascinating to explore the evolutionary stability of the cross-feeding studied here, particularly because the evolutionary stability may depend strongly upon the environmental context [36].

In this paper, we have focused on the interactions between two auxotrophic strains, each of which produces the amino acid needed by its partner. However, in principle, this cross-feeding mutualism can be invaded by other strains, the most relevant of which would be the double-producer (producing both tryptophan and leucine) and the non-producer (auxotroph for leucine and tryptophan). At least within the realm of our model, we predict that at intermediate amino acid concentrations the mutualism is non-invadable by either of these alternative strains (S5 Fig, S1 Information). However, at higher amino acid concentrations the non-producer is predicted to invade and coexist with the single producers (and, similarly, at lower amino acid concentrations the double producer is predicted to invade). It would be interesting to explore further the degree to which cross-feeding can stabilize the coexistence of multiple strains, particularly given the wide range of nutrients that can be shared in a microbial community.

It is also worth noting that the two strains used in our study were able to form an effective cross-feeding mutualism without ever having previously grown together, i.e., in the absence of coevolution. There is still considerable debate regarding whether mutualisms in natural microbial communities arise primarily from this sort of ecological fitting or via coevolution [19,25]. Laboratory experiments have demonstrated the stabilizing effects of coevolution on mutualism dynamics [11]. Regardless, we note that our mutualism dynamics are quite stable even in the absence of a period of coevolution.

One important feature of our mutualism is that the two strains are almost genetically identical. This means they have near-perfect niche overlap, which results in very strong competition between the two strains when amino acid concentrations are high. In many other mutualisms, the partners will have less niche overlap and will therefore experience less competition. Incorporating this in our model predicts that the degree of niche overlap will have a strong influence on the outcome of the interaction and the degree to which different environmental conditions will switch the nature of the interaction (S6 Fig). As perhaps expected, less niche overlap results in a larger range of parameters in which the species are mutualistic. Future studies in the field and in the laboratory will be needed to elucidate whether the wide range of interactions observed here is relevant for other mutualisms.

## Materials and Methods

### Strains

Both *S*. *cerevisiae* strains are from a W303 background and are genetically modified to cross-feed as described in [10]. The strains were adapted to growing with low amino acid supplementation through seven cycles of daily dilution (10X) and growth in 2 μM tryptophan and 32 μM leucine. In these cycles, populations consisting of ~100,000 to 500,000 cells underwent bottlenecks in which as few as 10,000 cells survived. Monoclonal lines from adapted strain were derived through plating on 1.5% agarose plates and were used for all experiments except for comparison with unadapted strains.

### Growth Media

Strains were grown in batch culture in synthetic medium consisting of Yeast Nitrogen Base (YNB, Sunrise Sciences), Complete Supplement Mixture lacking leucine and tryptophan (CSM-leu-trp, Sunrise Sciences), and 2% glucose. Synthetic medium was supplemented with varying amounts of amino acids as indicated in experiments. All daily dilution experiments were performed in BD Falcon 96-well flat bottom plates. Cells were grown in 200 μl batch culture at 30°C and mixed by a shaker rotating at 900 rpm. Plates were sealed with Bemis Laboratory Parafilm to prevent evaporation.

### Co-culture Experiments

At the start of each co-culture experiment, single colonies were grown for 24 h until saturation in 3 ml synthetic medium containing 100 μM tryptophan and 1,000 μM Leucine. They were then diluted by a factor of ten and grown for 4 h to prevent cells from being in stationary phase at the start of the experiment. Cells were spun down and washed three times to remove any excess amino acids. Leu^-^ and Trp^-^ cells were then mixed in appropriate ratios and seeded in BD Falcon 96-well flat bottom plates in 200 μl medium. A daily dilution cycle consisted of 23.5 h of growth, after which density was measured by spectrophotometry (Thermo Scientific VarioSkan Flash Multimode Reader) and relative abundance was measured by flow cytometry (Miltenyi MACSQuant VYB, minimum of 10,000 cells analyzed). Cultures were then diluted by a factor of ten into new 96-wells plates containing fresh medium

### Model Analysis

Figs 2 and 4 have been obtained by computing analytical formulae for the equilibrium point, eigenvalues, and eigenvectors of Eqs 1 and 2. Bifurcation analysis of the model is shown in S7 Fig. The analytical treatment has been carried out using a computer algebra system and can be found in the supplementary files (S2 Information). Simulated trajectories in the insets in Fig 4 have been obtained by Gillespie simulations [37] of the corresponding stochastic model of Eqs 1 and 2. The C code used for simulations is attached as supplementary material (S2 Information).

## Supporting Information

S1 Data Excel file containing the data used to create Figs 1–5 and S1–S5 Figs. (XLSX)

S1 Fig Relative fitness of Leu^-^ is lower than fitness of Trp^-^. To analyze relative fitness, we grew the strains in co-culture at saturating amino acid concentrations (200 μM tryptophan and 1600 μM leucine). With such high concentrations, additional amino acids provided through cross-feeding will give negligible benefits, thus enabling us to compare the intrinsic growth rate of the two strains. Co-cultures were started at 36 different combinations of initial density and abundance and grown for two cycles of daily dilution to reach carrying capacity. They were then grown for five additional days, and relative fitness was determined each day in every condition (S1 Information). Error bar indicates mean +- standard deviation (s.d.).
(PDF)

S2 Fig Individual tracks of co-culture abundances at different amino acid concentrations. Plots show individuals traces of experiments used for Fig 3. Co-cultures were grown at 16 different amino acid concentrations, ranging from 0 μM tryptophan and 0 μM leucine to 200 μM tryptophan and 1,600 μM leucine. Co-cultures were started at six different relative abundances and grown for seven cycles of daily dilution. Density of Trp- (green lines) and Leu- (red lines) was measured at the end of each day by spectrophotometry and flow cytometry.
(PDF)

S3 Fig Relative fitness as a function of relative abundance. To determine equilibria in co-cultures that had not yet reached saturation, we determined relative fitness as a function of the fraction of Leu^-^ cells. Co-cultures were grown for 2 d to reach carrying capacity, after which relative fitness was determined as described earlier (S1 Information). Relative fitness was then log transformed and plotted against the fraction of Leu^-^ cells at the start of that day. Bootstrapping was used to determine the equilibrium fraction, at which both strains have the same fitness (S1 Information).
(PDF)

S4 Fig Growth curves of *S*. *cerevisiae* strains before and after adaptation to low amino acids. Trp^-^ (A) and Leu^-^ (B) cells in exponential phase were seeded in 96-well flat bottom plates and incubated at 30°C for 32 h. Density was measured automatically every 10 min through spectrophotometry. Cells were either adapted (dashed lines) or not adapted (solid lines) to low amino acid concentrations by 7 d of growth-dilutions cycles with low amino acid supplementation. At the lowest amino acid concentrations (red lines), adapted strains grew much better than unadapted strains. At medium amino acid concentrations (blue lines), adapted strains still grew better than unadapted strains, although the unadapted Trp^-^ strain might still have reached the same carrying capacity. Interestingly, at high amino acid concentrations (green lines), unadapted strains grew better than adapted strain, suggesting a fitness trade-off between growth in low and high amino acid concentrations.
(PDF)

S5 Fig A cross-feeding mutualism can protect against invasion by other strains. Plot shows equilibrium density of simulations with four strains as a function of supplemented amino acids. Double producers (yellow line) are modelled to have a lower growth rate than single producers (red and green lines, equivalent to strain X and Y in Eqs 1 and 2), whereas non-producers (black line) have a higher growth rate than single producers (S1 Information). However, double producers produce both amino acids and thus do not benefit from extra amino acids. Non-producers produce no amino acids and are therefore completely dependent on amino acids provided in the medium or by other strains. Double-producers take over the population at low amino supplementation. They are not affected by the low concentration, whereas the other strains are severely hindered in growth. At high amino acid concentrations, the non-producer completely dominates the population. Cooperation provides little extra benefit over the nutrients already supplemented, while the cost of non-producing are a lot smaller. However, at intermediate amino acid concentrations, the mutualism is stable against invasion by both the double producer and the non-producer. Note that the equilibrium densities in this region are slightly different from equilibrium densities in Fig 2 because of a different normalization (S1 Information).
(PDF)

S6 Fig Smaller niche overlap results in a larger region of mutualistic interactions. Simulations were run to determine qualitative interaction as a function of supplemented amino acids (*a*) and niche overlap (*c*). Niche overlap was modelled as the degree to which each strain affects the carrying capacity of the other strain (S1 Information), with *c* = 1 being complete overlap and *c* = 0 being no niche overlap. The order of qualitative regimes remains unchanged, yet not all regimes are present with lower niche overlap, and smaller niche overlap generally results in a larger region of mutualistic interactions.
(PDF)

S7 Fig Different ecological regimes are revealed by bifurcation analysis of the model. Insets I–V are phase portraits of Eqs 1 and 2 obtained for various values of *a* (other parameters values given in the main text). Eigenvectors have normalized length. Inset I: (*a* = 0.08, extinction) all trajectories are attracted to global extinction. Inset II: (*a* = 0.09, obligatory mutualism) a stable co-culture equilibrium has emerged via a saddle-node bifurcation; trajectories either reach this equilibrium or go extinct. Inset III: *(a* = 0.13, obligatory/facultative mutualism) the saddle has moved to the *X* axis; all the co-cultures trajectories are now driven toward the co-culture equilibrium. Inset IV: (*a* = 0.23, competition) another saddle has been created on the *Y* axis; all co-cultures trajectories are driven toward the co-culture equilibrium. Inset V: (*a* = 0.9, competitive exclusion) the co-culture equilibrium has collapsed to the *X* axis.
(PDF)

S8 Fig Order of qualitative interactions is robust for changing death rates. Simulations were run to determine qualitative interactions as a function of supplemented amino acids (*a*) and death rate (δ). The model shifts through the same order of qualitative interactions in a large range of death rates.
(PDF)

S1 Information Supplementary information on data analysis and modelling. (PDF)

S2 Information Zip file containing the following files: “SM_analytical_treatment.nb” is a *Wolfram Mathematica* notebook file that contains the code used to generate Figs 2 and 4. “SM_analytical_treatment.cdf” contains the same code, but for the freely available software *CDF interactive player*. “SM_analytical_treatment.pdf” contains the PDF version and the figures of the same code. “Mutualism.c” contains the code written in ‘*C’* used to perform stochastic simulations used to generate Fig 4I–4V.
(ZIP)

## Abbreviations

Leu^-^: leucine auxotrophic strain
RFP: red fluorescent protein
s.d.: standard deviation
s.e.m.: standard error of the mean
Trp^-^: tryptophan auxotroph strain
YFP: yellow fluorescent protein
