# Correction: Resource Availability Modulates the Cooperative and Competitive Nature of a Microbial Cross-Feeding Mutualism

**DOI:** 10.1371/journal.pbio.1002606

**Published:** 2017-06-07

**Authors:** Tim A. Hoek, Kevin Axelrod, Tommaso Biancalani, Eugene A. Yurtsev, Jinghui Liu, Jeff Gore

The data associated with Figure 3 in S1 Data incorrectly shows the densities of the Trp- strain in co-culture in columns L-Q. It should display the densities of the Leu- strain in co-culture. Please view the correct S1 Data below.

## Supporting information

S1 Data Excel file containing the data used to create Figs 1–5 and S1–S5 Figs. (XLSX)
