# Supplementary figures and images for: Resource Availability Modulates the Cooperative and Competitive Nature of a Microbial Cross-Feeding Mutualism
Source: PLoS Biol. 2016 Aug 24;14(8):e1002540. doi: 10.1371/journal.pbio.1002540 (PMC4996419; doi:10.1371/journal.pbio.1002540)

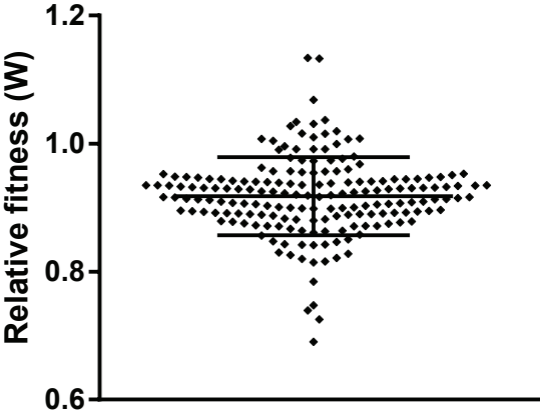

Supplement: S1 Fig — To analyze relative fitness, we grew the strains in co-culture at saturating amino acid concentrations (200 μM tryptophan and 1600 μM leucine). With such high concentrations, additional amino acids provided through cross-feeding will give negligible benefits, thus enabling us to compare the intrinsic growth rate of the two strains. Co-cultures were started at 36 different combinations of initial density and abundance and grown for two cycles of daily dilution to reach carrying capacity. They were then grown for five additional days, and relative fitness was determined each day in every condition (S1 Information). Error bar indicates mean +- standard deviation (s.d.). (PDF) [file pbio.1002540.s002.pdf]

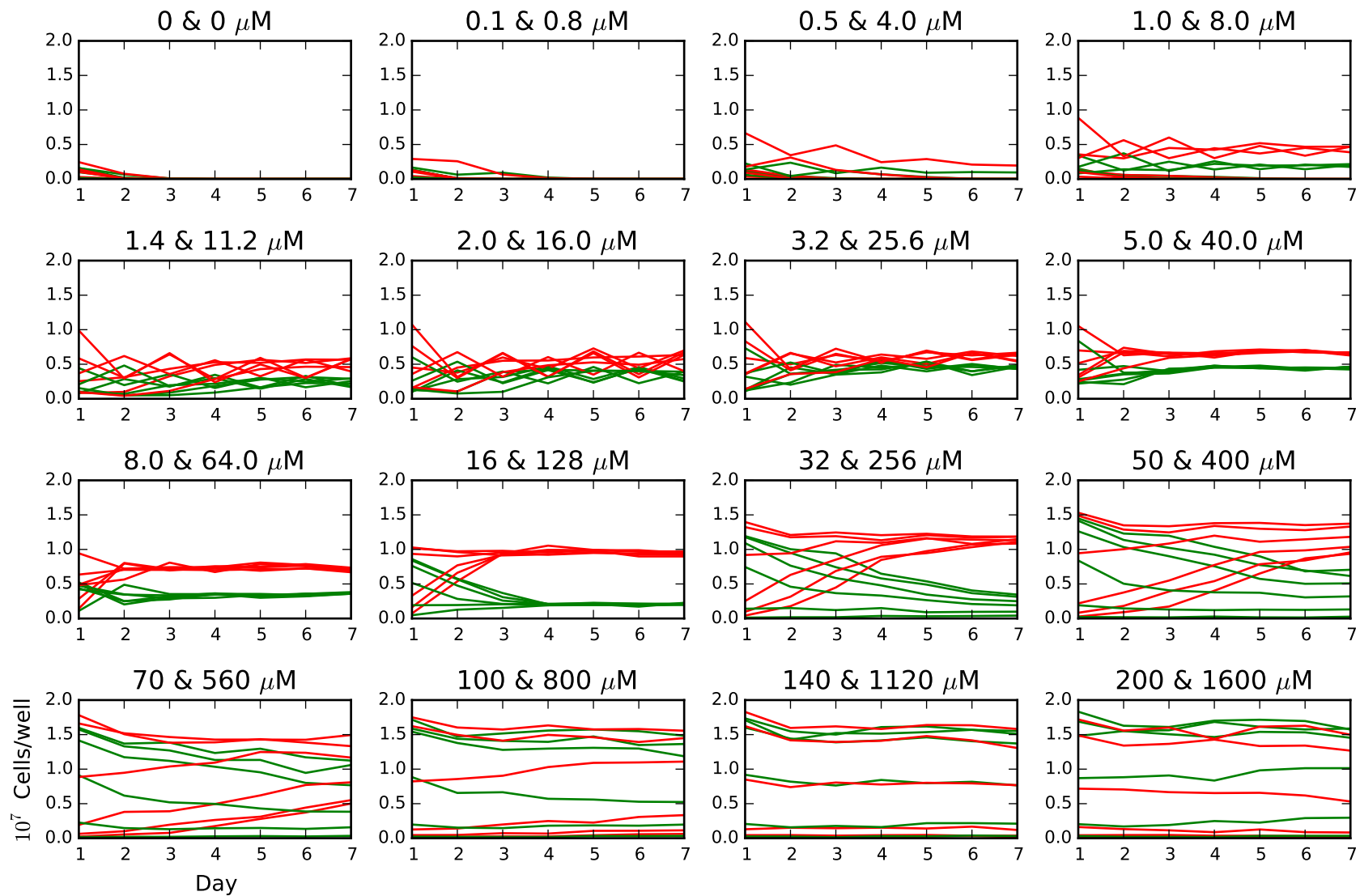

Supplement: S2 Fig — Plots show individuals traces of experiments used for Fig 3. Co-cultures were grown at 16 different amino acid concentrations, ranging from 0 μM tryptophan and 0 μM leucine to 200 μM tryptophan and 1,600 μM leucine. Co-cultures were started at six different relative abundances and grown for seven cycles of daily dilution. Density of Trp- (green lines) and Leu- (red lines) was measured at the end of each day by spectrophotometry and flow cytometry. (PDF) [file pbio.1002540.s003.pdf]

32 & 256  $\mu\text{M}$ 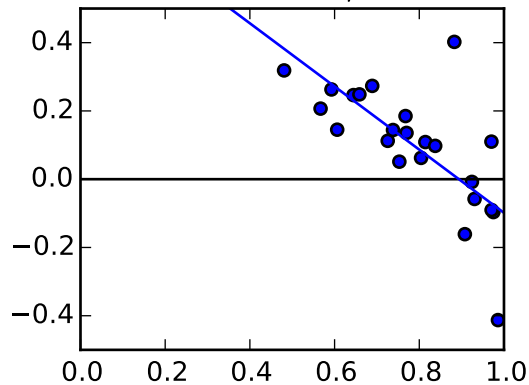50 & 400  $\mu\text{M}$ 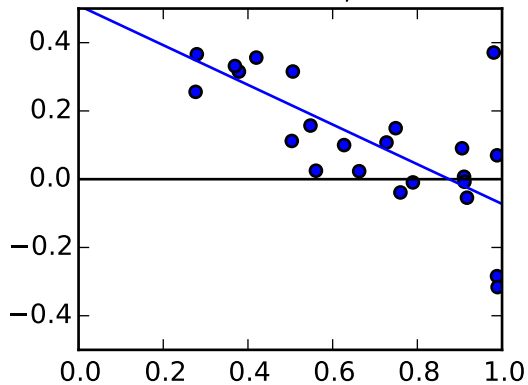70 & 560  $\mu\text{M}$ 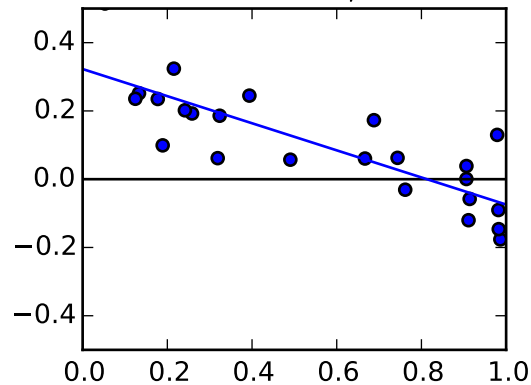100 & 800  $\mu\text{M}$ 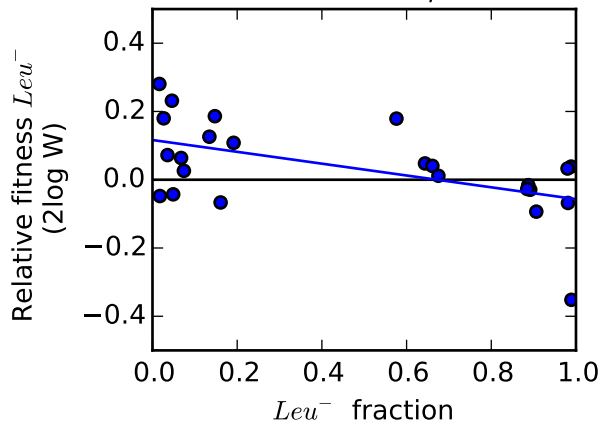140 & 1120  $\mu\text{M}$ 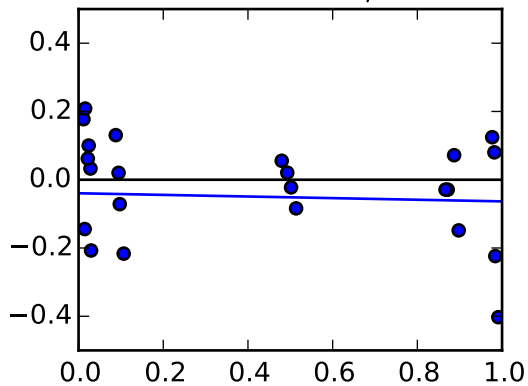200 & 1600  $\mu\text{M}$ 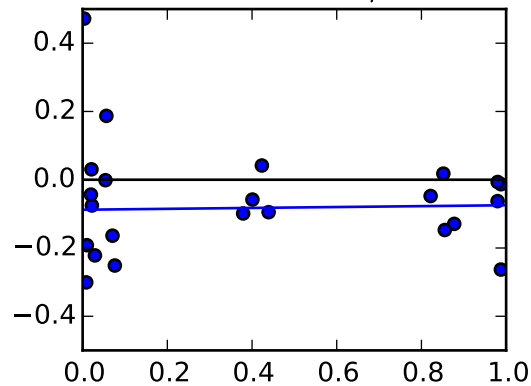

Supplement: S3 Fig — To determine equilibria in co-cultures that had not yet reached saturation, we determined relative fitness as a function of the fraction of Leu- cells. Co-cultures were grown for 2 d to reach carrying capacity, after which relative fitness was determined as described earlier (S1 Information). Relative fitness was then log transformed and plotted against the fraction of Leu- cells at the start of that day. Bootstrapping was used to determine the equilibrium fraction, at which both strains have the same fitness (S1 Information). (PDF) [file pbio.1002540.s004.pdf]

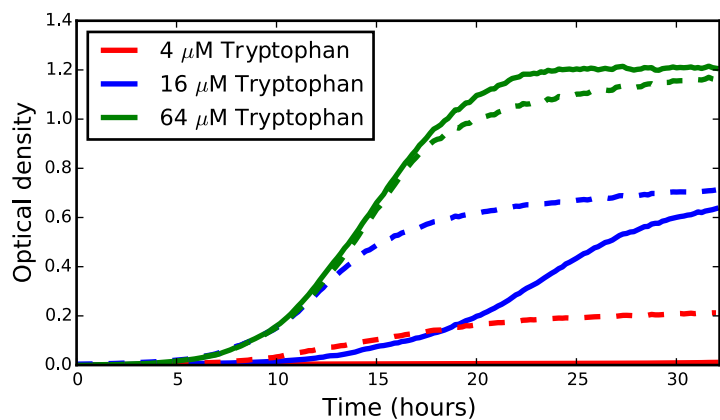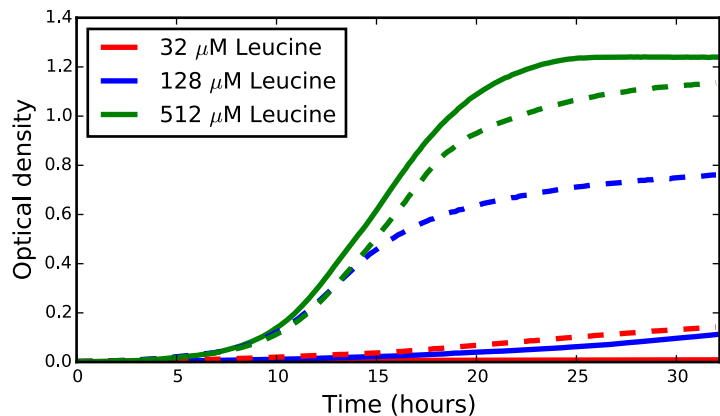

Supplement: S4 Fig — Trp- (A) and Leu- (B) cells in exponential phase were seeded in 96-well flat bottom plates and incubated at 30°C for 32 h. Density was measured automatically every 10 min through spectrophotometry. Cells were either adapted (dashed lines) or not adapted (solid lines) to low amino acid concentrations by 7 d of growth-dilutions cycles with low amino acid supplementation. At the lowest amino acid concentrations (red lines), adapted strains grew much better than unadapted strains. At medium amino acid concentrations (blue lines), adapted strains still grew better than unadapted strains, although the unadapted Trp- strain might still have reached the same carrying capacity. Interestingly, at high amino acid concentrations (green lines), unadapted strains grew better than adapted strain, suggesting a fitness trade-off between growth in low and high amino acid concentrations. (PDF) [file pbio.1002540.s005.pdf]

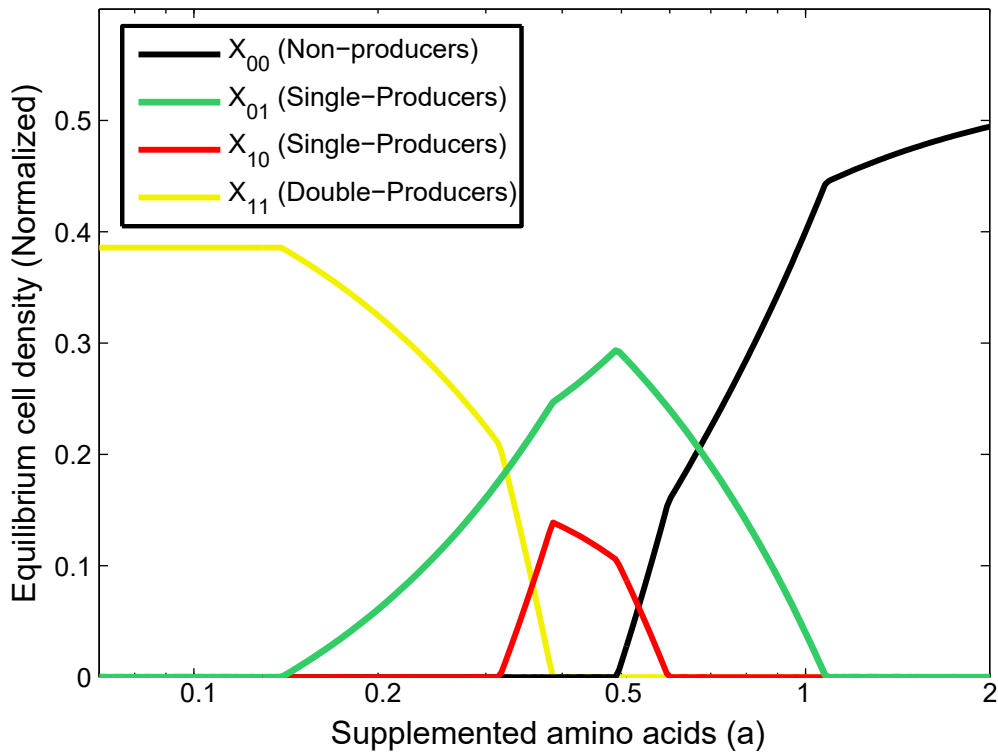

Supplement: S5 Fig — Plot shows equilibrium density of simulations with four strains as a function of supplemented amino acids. Double producers (yellow line) are modelled to have a lower growth rate than single producers (red and green lines, equivalent to strain X and Y in Eqs 1 and 2), whereas non-producers (black line) have a higher growth rate than single producers (S1 Information). However, double producers produce both amino acids and thus do not benefit from extra amino acids. Non-producers produce no amino acids and are therefore completely dependent on amino acids provided in the medium or by other strains. Double-producers take over the population at low amino supplementation. They are not affected by the low concentration, whereas the other strains are severely hindered in growth. At high amino acid concentrations, the non-producer completely dominates the population. Cooperation provides little extra benefit over the nutrients already supplemented, while the cost of non-producing are a lot smaller. However, at intermediate amino acid concentrations, the mutualism is stable against invasion by both the double producer and the non-producer. Note that the equilibrium densities in this region are slightly different from equilibrium densities in Fig 2 because of a different normalization (S1 Information). (PDF) [file pbio.1002540.s006.pdf]

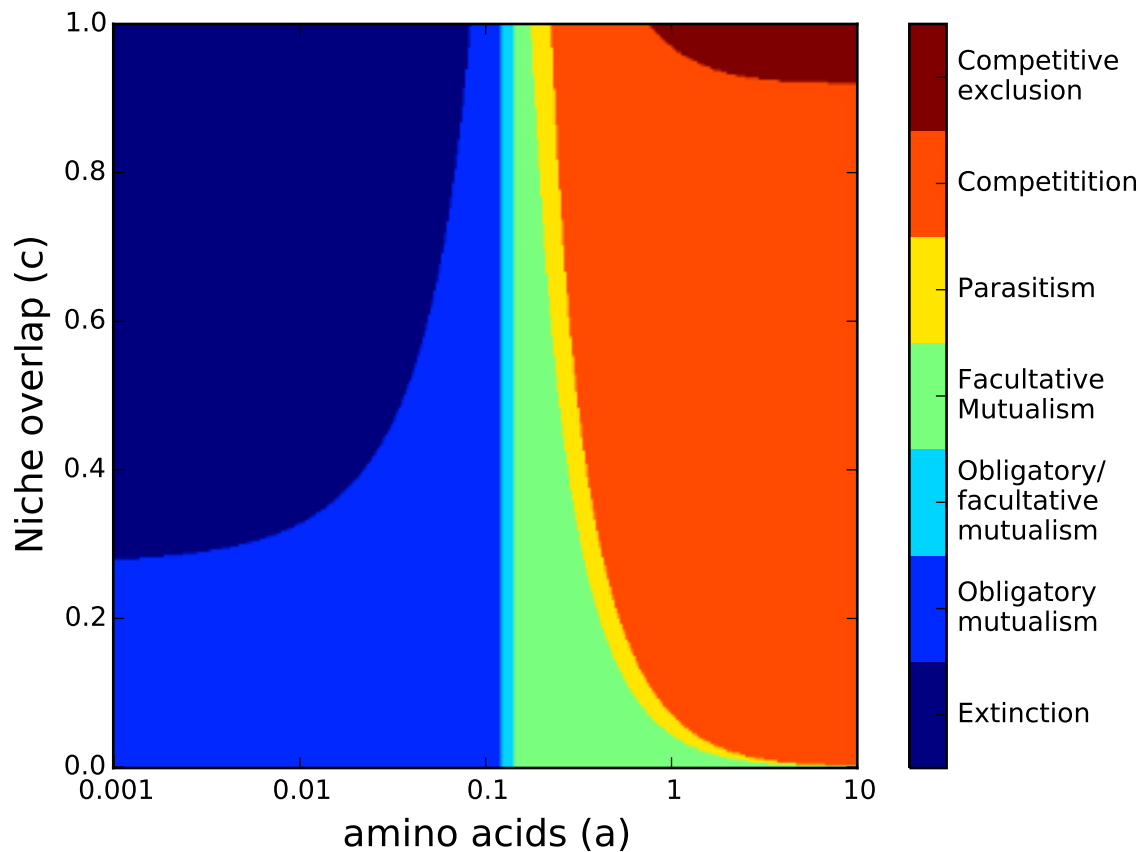

Supplement: S6 Fig — Simulations were run to determine qualitative interaction as a function of supplemented amino acids (a) and niche overlap (c). Niche overlap was modelled as the degree to which each strain affects the carrying capacity of the other strain (S1 Information), with c = 1 being complete overlap and c = 0 being no niche overlap. The order of qualitative regimes remains unchanged, yet not all regimes are present with lower niche overlap, and smaller niche overlap generally results in a larger region of mutualistic interactions. (PDF) [file pbio.1002540.s007.pdf]

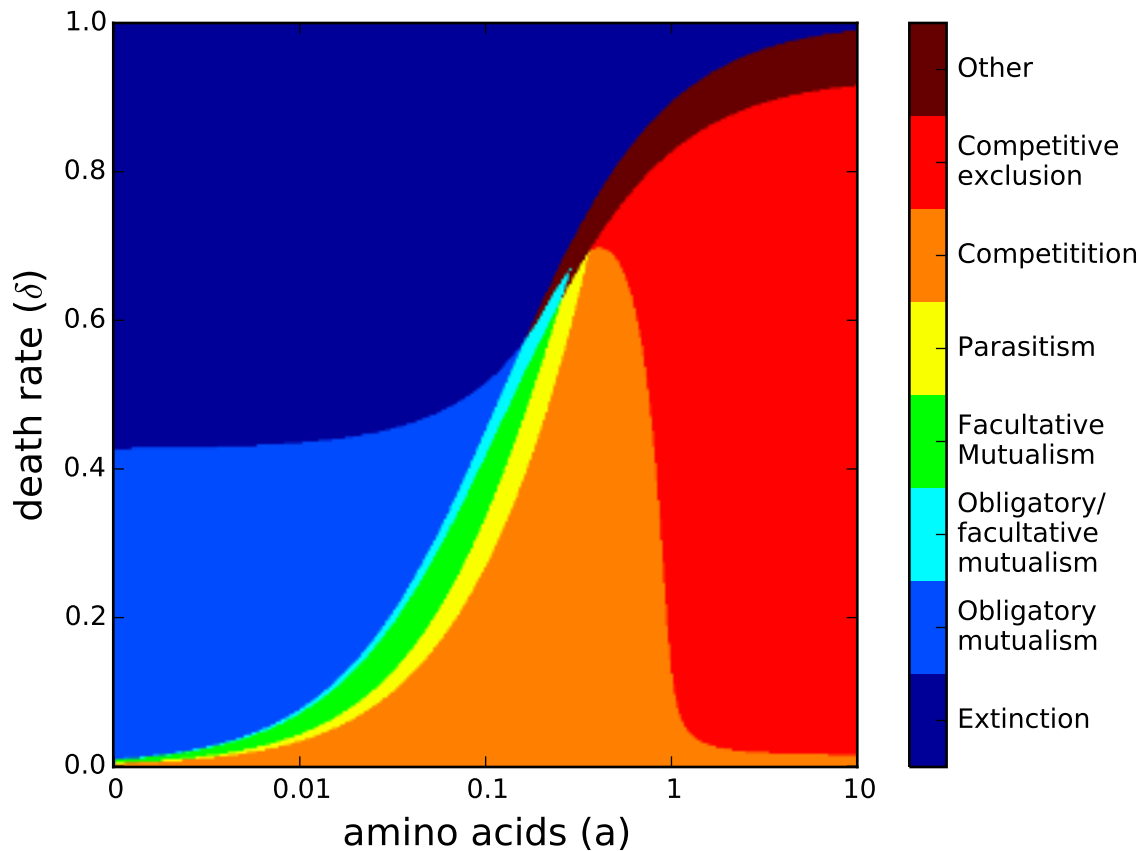

Supplement: S8 Fig — Simulations were run to determine qualitative interactions as a function of supplemented amino acids (a) and death rate (δ). The model shifts through the same order of qualitative interactions in a large range of death rates. (PDF) [file pbio.1002540.s009.pdf]
