# Supplementary material for: Resource Availability Modulates the Cooperative and Competitive Nature of a Microbial Cross-Feeding Mutualism
Source: PLoS Biol. 2016 Aug 24;14(8):e1002540. doi: 10.1371/journal.pbio.1002540 (PMC4996419; doi:10.1371/journal.pbio.1002540)
Supplement: S7 Fig — Insets I–V are phase portraits of Eqs 1 and 2 obtained for various values of a (other parameters values given in the main text). Eigenvectors have normalized length. Inset I: (a = 0.08, extinction) all trajectories are attracted to global extinction. Inset II: (a = 0.09, obligatory mutualism) a stable co-culture equilibrium has emerged via a saddle-node bifurcation; trajectories either reach this equilibrium or go extinct. Inset III: (a = 0.13, obligatory/facultative mutualism) the saddle has moved to the X axis; all the co-cultures trajectories are now driven toward the co-culture equilibrium. Inset IV: (a = 0.23, competition) another saddle has been created on the Y axis; all co-cultures trajectories are driven toward the co-culture equilibrium. Inset V: (a = 0.9, competitive exclusion) the co-culture equilibrium has collapsed to the X axis. (PDF) [file pbio.1002540.s008.pdf]

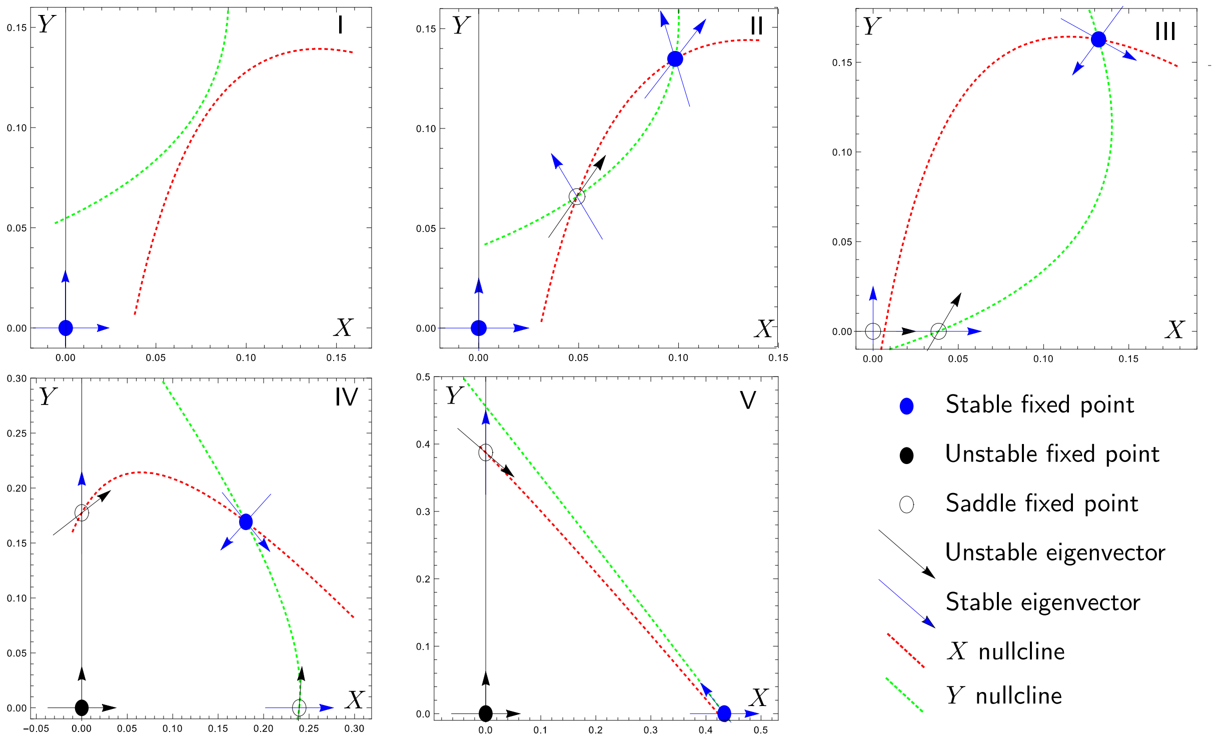

I
II
III
IV
V
Y
X
Stable fixed point
Unstable fixed point
Saddle fixed point
Unstable eigenvector
Stable eigenvector
X nullcline
Y nullcline
