# Supplementary material for: Resource Availability Modulates the Cooperative and Competitive Nature of a Microbial Cross-Feeding Mutualism
Source: PLoS Biol. 2016 Aug 24;14(8):e1002540. doi: 10.1371/journal.pbio.1002540 (PMC4996419; doi:10.1371/journal.pbio.1002540)
Supplement: S1 Information — (PDF) [file pbio.1002540.s010.pdf]

Supplementary information for "Resource availability modulates the cooperative and competitive nature of a microbial cross-feeding mutualism"

Hoek et al, *in consideration for publication in PLOS Biology* 2016

Calculating relative fitness

Our model assumes continuous culture (ie constant growth and dilution of the media), so for simplicity we focused on the mean growth rates over the course of each 24 hr growth dilution cycle. The relative relative fitness is then:

$W = r_1 / r_2,$ [3]

where r_1 is the mean growth rate of the less fit strain (Leu^-) and r_2 is the mean growth rate of the more fit strain (Trp^-). The mean growth rate was determined by comparing the cell densities at the start and end of each dilution cycle. Starting densities were calculated by dividing final density of the previous day by 10, since we diluted by a factor 10. The growth over the entire 24 hours is then:

$$N_1(t) = N_1(0)\, e^{r_1 t} \qquad [4]$$

$N_1(t)$ and $N_1(0)$ represent the densities at timepoint t or timepoint 0, respectively, and *t* represents the full 24 hours of growth and r_1 again represents the average growth rate over the course of the day. It is important to note that the cultures might be saturating, so the average growth rate will decrease if we let all the cultures grow for a longer period of time. However, to calculate the relative fitness we co-culture the strains, and the relative fitness is then:

$$W = \frac{\ln[\frac{N_1(t)}{N_1(0)}]}{\ln[\frac{N_2(t)}{N_2(0)}]} \qquad [5]$$

Importantly, we found that at high amino acid concentration (200 uM Trp & 1600 uM Leu) there was no detectable frequency dependence, yielding W = 0.93 (Fig S1).

Estimating equilibrium densities when cultures have not reached saturation.

Since co-cultures did not reach equilibrium density within 7 days at high amino acid concentrations (above 32 µM tryptophan & 256 µM leucine, figure 3), we estimated equilibrium densities by determining relative fitness as a function of relative abundances. We determined fitness of Leu^- as described above in

equation 3, and plotted the log(2) transformed fitness against the fraction of Leu^- cells. At a low fraction of Leu^- cells, Leu^- cells will have a fitness advantage over Trp^- cells because of the nutrients provide by Trp^-, and vice versa. However, when the strains are at equilibrium fraction they will have equal fitness (since abundance does not change anymore). We used bootstrapping to calculate at which fraction the cells had equal fitness. We ran a simulation in which we randomly resampled our data (with replacement) 100,000 times. In each resampling, we fitted a first degree polynomial and calculated the Leu^- fraction at which fitness was equal. Equilibrium fractions below zero were rounded up to zero, and fractions above one were rounded down to one. We then calculated the mean equilibrium fraction (+- s.e.m.) of these simulations.

To estimate the equilibrium density of both strains, we combined data of equilibrium fraction and equilibrium total density. We assumed that at these high amino acid concentrations, co-cultures will reach equilibrium total density within a few days, and therefore calculated the variation in total density similar to saturated co-cultures (mean +- s.e.m. of the means of six conditions over the last three days). Equilibrium density of individual strains was calculated by multiplying equilibrium density with equilibrium fraction. Standard error of the mean of densities of individual strains was calculated by the formula:

$$SE_f \approx \sqrt{B^2 SE_a^2 + A^2 SE_b^2} \qquad [6]$$

In which SE_f is the standard error of the mean of the density of one strain, A is the total density, B is the equilibrium fraction of that strain, and SE_a and SE_b are the s.e.m. of total density and equilibrium fraction. The standard deviations calculated with equations 6 are depicted in figure 3.

Modelling of interactions between four species.

To analyze the behavior of our system in the presence of other species, we extended our model to a four-species system. This system includes the two initial cross-feeding strains, which are given in this model by X_{01} and X_{10}. The strain X_{01} (which is Trp^- or strain X) does not produce the first nutrient (tryptophan), but does produce the second nutrient (leucine), while strain X_{10} (Leu^- or Y) produces the first but not the

second nutrient. The two new strains are X_{11}, which produces both nutrients, and X_{00}, which produces no nutrients. Equilbrium densities are calculated with the following equations:

$$\frac{dX_{00}}{dt} = X_{00}\left(\frac{X_{10} + X_{11} + a}{X_{10} + X_{11} + a + \kappa}\right)\frac{\beta X_{01} + X_{11} + a}{\beta X_{01} + X_{11} + a + \kappa}(1 - X_{00} - X_{01} - X_{10} - X_{11}) - \delta X_{00} \quad [7]$$

$$\frac{dX_{01}}{dt} = (1 - c_1)X_{01}\left(\frac{X_{10} + X_{11} + a}{X_{10} + X_{11} + a + \kappa}\right)(1 - X_{00} - X_{01} - X_{10} - X_{11}) - \delta X_{01} \quad [8]$$

$$\frac{dX_{10}}{dt} = (1 - c_2)X_{10}\left(\frac{\beta X_{01} + X_{11} + a}{\beta X_{01} + X_{11} + a + \kappa}\right)(1 - X_{00} - X_{01} - X_{10} - X_{11}) - \delta X_{10} \quad [9]$$

$$\frac{dX_{00}}{dt} = (1 - c_1 - c_2)X_{00}(1 - X_{00} - X_{01} - X_{10} - X_{11}) - \delta X_{11} \quad [10]$$

The growth rate of each strain increases with the abundance of the amino acids they cannot produce. Amino acids can be acquired from supplementation (a) or from mutualist partners (X_{11}, X_{10} or X_{01}), and again saturate via a Michaelis-Menten form with the same scaling constant (kappa = 0.12). We also maintained the unequal contribution to the mutualism (β = 2). Since all species have the same nutrient requirements apart from leucine and tryptophan, they saturate at a carrying capacity that is normalized to 1. The cost of producing nutrients is modelled by a cost that reduces the growth rate. The growth rate of the non-producer, which does not produce amino acids and thus has no costs, is normalized to 1. We modelled a different cost of producing each nutrient (c_1 = 0.10 and c_2 = 0.1675), so that the growth rate of strain X_{10} remains 0.925 times that of strain X_{01} (0.8325 / 0.90 = 0.925). Since the double-producer makes both nutrients, it's growth rate is reduced by c_1 and c_2 (1 – 0.10 – 0.1675 = 0.7325). To compensate for the reduced growth rates compared to our original model, we normalized the death rate by a factor (1 – c_1 = 0.9), resulting in a death rate of δ = 0.45.

Modelling niche overlap

In our standard model (eq. 1 and 2), we assumed complete niche overlap, since both yeast strains are identical except for a few genetic modifications. This means they require the exact same nutrients (apart

from leucine and tryptophan), and their growth is thus limited equally by saturation of either strain, which we modelled as *(1 – X – C)*. However, if the two strains do not require the exact same nutrients, e.g. when one strain grows on glucose and the other strain grows on sucrose, there will not be complete niche overlap. In this case, one strain could still grow even when the other strain has reached carrying capacity. We modelled this by introducing niche overlap 'c' into the saturation term. This way, strain *X* saturates by *(1 – X – cY),* and strain *Y* saturates through *(1 – cX – Y)*:

$$\frac{dX}{dt} = r_x X \left(\frac{Y+a}{Y+a+\kappa}\right)(1 - X - cY) - \delta X \qquad [11]$$

$$\frac{dY}{dt} = r_y Y \left(\frac{X+a}{X+a+\kappa}\right)(1 - cX - Y) - \delta Y \qquad [12]$$

Qualitative interactions were determined by running simulations with different values of *a* and *c*. Simulations were started with abundances of both strains above carrying capacity. Note that for cross-species mutualisms niche overlap will typically not be complete.

Resource explicit modelling

Equations [1] and [2] present a simplified, phenomenological description of two species interacting via cross-feeding. In particular, in these equations we do not model the amino acid concentrations explicitly. These equations can be viewed from a purely phenomenological perspective, but in addition they can be derived rigorously from a resource-explicit model in some limiting cases. With amino acid concentrations modeled explicitly we have:

$$\frac{dX}{dt} = r_x X \frac{a_y}{a_y + k}(1 - X - Y) - \delta X$$

$$\frac{dY}{dt} = r_y Y \frac{a_x}{a_x + k}(1 - X - Y) - \delta Y$$

$$\dot{a}_x = g + \alpha_x X - \gamma_x Y - d \cdot a_x$$

$$\dot{a}_y = g + \alpha_y Y - \gamma_y X - d \cdot a_y$$

g : Rate by which the amino acid is supplied in the media.

$\alpha_x(\alpha_y)$: Amino acid production rate by X (or Y).

$\gamma_x(\gamma_y)$: Amino acid consumption rate by Y (or X).

d : Amino acid degradation/dilution rate in environment

k : Michaelis-Menten constant describing saturation of nutrient benefits for species growth

$r_x(r_y)$: Growth rates of species X (or Y)

δ : Species death rate

In the limit that amino acid consumption by the species is much smaller than degradation/dilution of the amino acid, the resource-explicit form above simplifies to a resource-implicit form based on a "fast equilibrium approximation" of nutrient and species dynamics:

$$\frac{dX}{dt} = r_x X \frac{Y + g/\alpha_y}{Y + g/\alpha_y + k \cdot d/\alpha_y}(1 - X - Y) - \delta \cdot X$$

$$\frac{dY}{dt} = r_y Y \frac{\frac{\alpha_x}{\alpha_y} X + g/\alpha_y}{\frac{\alpha_x}{\alpha_y} X + g/\alpha_y + k \cdot d/\alpha_y}(1 - X - Y) - \delta \cdot Y$$

Comparing with Equations [1] and [2]:

$$\frac{dX}{dt} = r_x X \frac{Y + a}{Y + a + \kappa}(1 - X - Y) - \delta X$$

$$\frac{dY}{dt} = r_y Y \frac{\beta X + a}{\beta X + a + \kappa}(1 - X - Y) - \delta Y$$

we get a relation of simplified parameters:

$$a = g/\alpha_y \in [0,1]$$

$$\beta = \alpha_x/\alpha_y = 2$$

$$\kappa = k \cdot d/\alpha_y = 0.12$$

$$r_x = 1, r_y = 0.925$$

$$\delta = 0.5$$

We verified in simulations that the resource-explicit model produces the same species dynamics as the resource-implicit model when in reasonable parameter ranges (figure X1). Additionally, when non-negligible values for amino acid consumption are used, the model predicts a shift through the same qualitative interaction regimes, indicating that amino acid consumption rates do not alter the predictions of our model. Furthermore, we tested whether the speed by which amino acid concentrations equilibrate affect the overall behavior of the resource-explicit model by varying amino acid production (α_x/α_y) and amino acid degradation/dilution (d) rates. Our model was robust for changes in these rates, and amino acid concentrations and population densities equilibrated to the results in figure 1 in a large range of parameters.

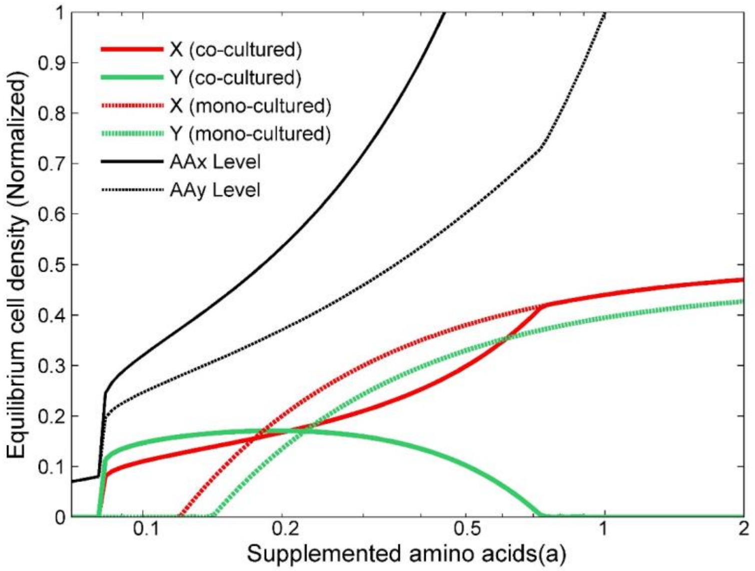

Figure X1. Results of simulations with a resource-explicit model. Equilibrium densities of monocultures (dotted lines) and co-cultures (full lines) are shown as a function of supplemented amino acid. Black lines show equilibrium amino acid concentrations.
