# Supplementary material for: Resource Availability Modulates the Cooperative and Competitive Nature of a Microbial Cross-Feeding Mutualism
Source: PLoS Biol. 2016 Aug 24;14(8):e1002540. doi: 10.1371/journal.pbio.1002540 (PMC4996419; doi:10.1371/journal.pbio.1002540)
Supplement: S2 Information — “SM_analytical_treatment.cdf” contains the same code, but for the freely available software CDF interactive player. “SM_analytical_treatment.pdf” contains the PDF version and the figures of the same code. “Mutualism.c” contains the code written in ‘C’ used to perform stochastic simulations used to generate Fig 4I–4V. (ZIP) [file pbio.1002540.s011.zip › S2Information/SM_analytical_treatment.pdf]

Supplementary Material for the manuscript “Environmental quality modulates the cooperative and competitive nature of a microbial cross-feeding mutualism”
This *Mathematica* file contains analytical calculations used to derive fixed point concentrations and corresponding eigenvalues/eigenvectors.
Please address any queries about this SM to Tommaso Biancalani - <tommasob@mit.edu>

Cross-feeding mutualism ODEs model - Analytical treatment

Co-culture and monoculture equations

In[608]:=

$$\texttt{xRHS} = \texttt{r1}\,X\,\frac{Y+a}{Y+a+k}\,(1-X-Y)-\delta\,X;\quad \texttt{yRHS} = \texttt{r2}\,Y\,\frac{\beta X+a}{\beta X+a+k}\,(1-X-Y)-\delta\,Y;$$

$$\texttt{mRHS} = r\,m\,\frac{a}{a+k}\,(1-m)-\delta\,m;$$

Analysis of monoculture equation

Fixed points

In[610]:=
```
fpsm = Simplify[Solve[{mRHS == 0}, {m}, Reals]];
```

In[611]:=
```
fpsm // Length
```

Out[611]= 2

In[612]:=
```
fpsm // Normal
```

Out[612]=

$$\left\{\{m\to 0\},\ \left\{m\to \frac{a\,r-a\,\delta-k\,\delta}{a\,r}\right\}\right\}$$

In[613]:=
```
fp1mval = (fpsm[[1]] // Normal) // Simplify;
fp2mval = (fpsm[[2]] // Normal) // Simplify;
```

Stability domains of the two fixed points

In[615]:=
```
Dm1 = Simplify[D[mRHS, m]] /. fp1mval // Simplify;
Dm2 = Simplify[D[mRHS, m]] /. fp2mval // Simplify;
```

In[617]:=
```
fp1mStabNodeDom = Reduce[{Dm1 < 0, a > 0, k > 0, 0 < δ < 1, r > 0}, a]
```

Out[617]=

$$\left(0<r\le 1\ \&\&\ \left(\left(0<\delta<r\ \&\&\ k>0\ \&\&\ 0<a<-\frac{k\,\delta}{-r+\delta}\right)\ ||\ (r\le\delta<1\ \&\&\ k>0\ \&\&\ a>0)\right)\right)\ ||\ \left(r>1\ \&\&\ 0<\delta<1\ \&\&\ k>0\ \&\&\ 0<a<-\frac{k\,\delta}{-r+\delta}\right)$$

In[618]:=
```
fp2mStabNodeDom = Reduce[{Dm2 > 0, a > 0, k > 0, 0 < δ < 1, r > 0}, a]
```

Out[618]=

$$\left(0<r\le 1\ \&\&\ \left(\left(0<\delta<r\ \&\&\ k>0\ \&\&\ 0<a<-\frac{k\,\delta}{-r+\delta}\right)\ ||\ (r\le\delta<1\ \&\&\ k>0\ \&\&\ a>0)\right)\right)\ ||\ \left(r>1\ \&\&\ 0<\delta<1\ \&\&\ k>0\ \&\&\ 0<a<-\frac{k\,\delta}{-r+\delta}\right)$$

Supplemented a.a. thresholds for monoculture instability

In[619]:=
```
ac1X = k δ/(r1 - δ); ac1Y = k δ/(r2 - δ);
```

Parameter values

In[620]:=
```
r1 = 1; r2 = 1 - ϵ; β = 2; δ = 1/2; k = 3/25; ϵ = 3/40;
```

Fixed points and Jacobian matrices of co-culture equations

Fixed points expressions

In[621]:=
```
fps = Simplify[Solve[{xRHS == 0, yRHS == 0}, {X, Y}, Reals]];
```

In[622]:=
```
fps // Length
```

Out[622]= 6

In[623]:=
```
fp1val = (fps[[1]] // Normal) // Simplify;
fp2val = (fps[[2]] // Normal) // Simplify;
fp3val = (fps[[3]] // Normal) // Simplify;
fp4val = FullSimplify[(fps[[4]] // Normal), Assumptions → {a > 0}];
fp5val = FullSimplify[(fps[[5]] // Normal), Assumptions → {a > 0}];
fp6val = FullSimplify[(fps[[6]] // Normal), Assumptions → {a > 0}];
```

Jacobian matrices evaluated on the fixed points

In[629]:=
```
J = ( D[xRHS, X]  D[xRHS, Y]
      D[yRHS, X]  D[yRHS, Y] ) // FullSimplify;
```

In[630]:=
```
J1 = J /. fp1val;
det1 = FullSimplify[Det[J1], Assumptions → {a > 0}];
tr1 = FullSimplify[Tr[J1], Assumptions → {a > 0}];
```

In[633]:=
```
J2 = FullSimplify[(J /. fp2val), Assumptions → {a > 0}];
det2 = FullSimplify[Det[J2], Assumptions → {a > 0}];
tr2 = FullSimplify[Tr[J2], Assumptions → {a > 0}];
```

In[636]:=
```
J3 = FullSimplify[(J /. fp3val), Assumptions → {a > 0}];
det3 = FullSimplify[Det[J3], Assumptions → {a > 0}];
tr3 = FullSimplify[Tr[J3], Assumptions → {a > 0}];
```

In[639]:=
```
J4 = FullSimplify[(J /. fp4val), Assumptions → {a > 0}];
det4 = FullSimplify[Det[J4], Assumptions → {a > 0}];
tr4 = FullSimplify[Tr[J4], Assumptions → {a > 0}];
```

```
J5 = FullSimplify[(J /. fp5val), Assumptions → {a > 0}];
det5 = FullSimplify[Det[J5], Assumptions → {a > 0}];
tr5 = FullSimplify[Tr[J5], Assumptions → {a > 0}];
```

In[645]:=
```
J6 = FullSimplify[(J /. fp6val), Assumptions → {a > 0}];
det6 = FullSimplify[Det[J6], Assumptions → {a > 0}];
tr6 = FullSimplify[Tr[J6], Assumptions → {a > 0}];
```

Linear stability analysis of fixed point #1

FP1 corresponds to extinction

In[648]:=
```
fp1val
```

Out[648]= $\{X \to 0, Y \to 0\}$

Parameter domain for which FP1 is respectively a stable node and a saddle

In[649]:=
```
fp1StabNodeDom = Reduce[{det1 > 0, tr1 < 0, tr1^2 - 4 det1 > 0, a > 0}, a]
```

Out[649]= $0 < a < \frac{3}{25}$

In[650]:=
```
fp1SdleDom = Reduce[{det1 < 0, a > 0}, a]
```

Out[650]= $\frac{3}{25} < a < \frac{12}{85}$

Instability domain

In[651]:=
```
fp1UnstNodeDom = Reduce[{det1 > 0, tr1 > 0, a > 0}, a]
```

Out[651]= $a > \frac{12}{85}$

Linear stability analysis of fixed point #2 and #3

FP2 and FP3 correspond to partial extinctions

In[652]:=
```
fp2val
fp3val
```

Out[652]= $\left\{X \to 0, Y \to \frac{-12 + 85 a}{185 a}\right\}$

Out[653]= $\left\{X \to \frac{1}{2} - \frac{3}{50 a}, Y \to 0\right\}$

They have physical meaning when the concentrations take positive values

In[654]:=
```
fp2Dom = Reduce[{(Y /. fp2val) > 0, a > 0}, Reals]
fp3Dom = Reduce[{(X /. fp3val) > 0, a > 0}, Reals]
```

Out[654]= $a > \frac{12}{85}$

Out[655]= $a > \frac{3}{25}$

Parameter domains in which FP2/FP3 is a saddle

In[656]:=
```
fp2SdleDom = Reduce[{det2 < 0, (Y /. fp2val) > 0, a > 0}, a, Reals]
```

Out[656]= $a > \frac{12}{85}$

In[657]:=
```
fp3SdleDom = Reduce[{det3 < 0, a > 0, (X /. fp3val) > 0}, a, Reals] // N
```
Out[657]= $0.122692 < a < 0.73276$

Parameter domain in which FP3 is a stable node

In[658]:=
```
fp3StabNodeDom = Reduce[{det3 > 0, tr3 < 0, a > ac1X, (X /. fp3val) > 0}] // N
```
Out[658]= $0.12 < a < 0.122692 \,||\, a > 0.73276$

Linear stability analysis of fixed points #4, #5 and #6

These three fixed points appear as roots of a cubic polynomial. One of these points has real coordinates, whereas the other two may have complex conjugate coordinates.

In[659]:=
```
FullSimplify[fp4val, Assumptions → {a > 0}]
```
Out[659]=
```
{X → Root[96 - 620 a - 536 a^2 - 925 a^3 + (-1240 + 2296 a - 2775 a^2) #1 + 6736 #1^2 + 3700 #1^3 &, 1],
 Y →
  -Root[96 - 620 a - 536 a^2 - 925 a^3 + (-1240 + 2296 a - 2775 a^2) #1 + 6736 #1^2 + 3700 #1^3 &, 1] +
   1/185 (85 - 12 / (a + 2 Root[96 - 620 a - 536 a^2 - 925 a^3 +
          (-1240 + 2296 a - 2775 a^2) #1 + 6736 #1^2 + 3700 #1^3 &, 1]))}
```

In[660]:=
```
FullSimplify[fp5val, Assumptions → {a > 0}]
```
Out[660]=
```
{X → Root[96 - 620 a - 536 a^2 - 925 a^3 + (-1240 + 2296 a - 2775 a^2) #1 + 6736 #1^2 + 3700 #1^3 &, 2],
 Y →
  -Root[96 - 620 a - 536 a^2 - 925 a^3 + (-1240 + 2296 a - 2775 a^2) #1 + 6736 #1^2 + 3700 #1^3 &, 2] +
   1/185 (85 - 12 / (a + 2 Root[96 - 620 a - 536 a^2 - 925 a^3 +
          (-1240 + 2296 a - 2775 a^2) #1 + 6736 #1^2 + 3700 #1^3 &, 2]))}
```

In[661]:=
```
FullSimplify[fp6val, Assumptions → {a > 0}]
```
Out[661]=
```
{X → Root[96 - 620 a - 536 a^2 - 925 a^3 + (-1240 + 2296 a - 2775 a^2) #1 + 6736 #1^2 + 3700 #1^3 &, 3],
 Y →
  -Root[96 - 620 a - 536 a^2 - 925 a^3 + (-1240 + 2296 a - 2775 a^2) #1 + 6736 #1^2 + 3700 #1^3 &, 3] +
   1/185 (85 - 12 / (a + 2 Root[96 - 620 a - 536 a^2 - 925 a^3 +
          (-1240 + 2296 a - 2775 a^2) #1 + 6736 #1^2 + 3700 #1^3 &, 3]))}
```

These three fixed points take real coordinate values when the following condition is satisfied

In[662]:=
```
pol = 96 - 620 a - 536 a^2 - 925 a^3 + (-1240 + 2296 a - 2775 a^2) x + 6736 x^2 + 3700 x^3;
{pold, polc, polb, pola} = CoefficientList[pol, x];
polΔ = 18 pola polb polc pold - 4 polb^3 pold + polb^2 polc^2 - 4 pola polc^3 - 27 pola^2 pold^2;
```

In[665]:=
```
Reduce[{polΔ > 0, a > 0}, a] // N
```
Out[665]= $a > 0.0823694$

The condition defines our threshold

In[666]:=
```
ac6a = a /. Solve[polΔ == 0, a][[4]] // N
```
Out[666]=
```
0.0823694
```

FP4 is a saddle with non-physical coordinates

In[667]:=
```
Reduce[{det4 < 0, a > 0}]
Reduce[{(X /. fp4val) < 0, a > 0}]
```
Out[667]= $a > 0$

Out[668]= $a > 0$

FP6 is a stable node for a certain parameter regime. This FP corresponds to our non-trivial equilibrium

In[669]:=
```
fp6StabNodeDom = Reduce[{det6 > 0, tr6 < 0,
    tr6^2 - 4 det6 > 0, (X /. fp6val) > 0, (Y /. fp6val) > 0, a > 0}, Reals] // N
```
Out[669]= $0.0823694 < a < 0.73276$

After the threshold is crossed, FP6 becomes a saddle that pushes toward the new stable FP. This threshold initiates the competitive exclusion regime

In[670]:=
```
fp6SdleDom = Reduce[{det6 < 0, a > 0}, Reals] // N
```
Out[670]= $a > 0.73276$

In[671]:=
```
ac6b = 0.7327601214865619`;
```

FP5 is either a saddle or takes negative concentration values.

In[672]:=
```
Reduce[{det5 > 0, (X /. fp5val) > 0, (Y /. fp5val) > 0, a > 0}, Reals] // N
```
Out[672]=
```
False
```

Thresholds related to mono-culture system

In[673]:=
```
fpmX = (m /. fp2mval) /. r → r1;
fpmY = (m /. fp2mval) /. r → r2;
```

fp2mval is the non-trivial FP of the monoculture

In[675]:=
```
fp2mval
```
Out[675]= $\left\{m \to \frac{-\frac{3}{50} - \frac{a}{2} + a\,r}{a\,r}\right\}$

We study when the co-culture non trivial FP exceeds the monoculture FP

In[676]:=
```
Solve[fpmX == (X /. fp6val), a, Reals] // N
Solve[fpmY == (Y /. fp6val), a, Reals] // N
```
Out[676]= $\{\{a \to 0.73276\}, \{a \to -1.15778\}, \{a \to 0.174196\}\}$

Out[677]= $\{\{a \to -0.732304\}, \{a \to 0.223848\}\}$

This defines our thresholds:

In[678]:=
```
acmX = 0.17419567244005071`;
acmY = 0.2238476064190428`;
```

Cross-feeding mutualism ODEs model - Figures

Init

In[680]:=
```
pwSplit[_[pairs : {{_, _} ..}]] := Piecewise[{#}, Indeterminate] & /@ pairs

pwSplit[_[pairs : {{_, _} ..}, expr_]] :=
 Append[pwSplit@{pairs}, pwSplit@{{{expr, Nor @@ pairs[[All, 2]]}}}]
```

In[682]:=
```
ac1XGra = Line[{{Log[ac1X] // N, -.6}, {Log[ac1X] // N, 1}}];
ac1YGra = Line[{{Log[ac1Y], -.6}, {Log[ac1Y], 1}}];
ac6aGra = Line[{{Log[ac6a], -.6}, {Log[ac6a], 1}}];
ac6bGra = Line[{{Log[ac6b], -.6}, {Log[ac6b], 1}}];
acmXGra = Line[{{Log[acmX], -.6}, {Log[acmX], 1}}];
acmYGra = Line[{{Log[acmY], -.6}, {Log[acmY], 1}}];
```

In[688]:=
```
ac1X // N
ac1Y // N
ac6a // N
ac6b // N
```

Out[688]= 0.12

Out[689]= 0.141176

Out[690]= 0.0823694

Out[691]= 0.73276

In[692]:=
```
fp1XStab = Piecewise[{{(X /. fp1val), fp1StabNodeDom}}, Null];
fp1XSdle = Piecewise[{{(X /. fp1val), fp1SdleDom}}, Null];
fp1XUnst = Piecewise[{{(X /. fp1val), fp1UnstNodeDom}}, Null];
fp3XSdle = Piecewise[{{X /. fp3val, fp3SdleDom}}, Null];
fp3XNode = Piecewise[{{X /. fp3val, fp3StabNodeDom}}, Null];
fp6XNode = Piecewise[{{X /. fp6val, fp6StabNodeDom}}, Null];
fp6XSdle = Piecewise[{{X /. fp6val, fp6SdleDom}}, Null];
```

In[699]:=
```
fp1YStab = Piecewise[{{(Y /. fp1val), fp1StabNodeDom}}, Null];
fp1YSdle = Piecewise[{{(Y /. fp1val), fp1SdleDom}}, Null];
fp1YUnst = Piecewise[{{(Y /. fp1val), fp1UnstNodeDom}}, Null];
fp6YNode = Piecewise[{{Y /. fp6val, fp6StabNodeDom}}, Null];
fp6YSdle = Piecewise[{{Y /. fp6val, fp6SdleDom}}, Null];
```

In[704]:=
```
{aMin, aMax} = {.05, 1};
```

In[705]:=
```
thresh = {ac1XGra, ac1YGra, ac6aGra, ac6bGra, acmXGra, acmYGra};
```

Figure: Equilibria vs Supplemented Amino Acids

In[706]:=
```
Xmgra = LogLinearPlot[{fpmX}, {a, aMin, aMax},
   PlotStyle → {Directive[Green, Dashed]}, Frame → True, PlotRange → {0, 0.45}];
Ymgra = LogLinearPlot[{fpmY}, {a, aMin, aMax}, PlotStyle → {Directive[Red, Dashed]},
   Frame → True, PlotRange → {0, 0.45}];
```

```
In[708]:= Xgra = LogLinearPlot[{fp3XNode, fp6XNode}, {a, aMin, aMax},
    PlotStyle → {Directive[Green, Thick]}, Frame → True, PlotRange → {0, 0.7}];
  Ygra = LogLinearPlot[{fp6YNode}, {a, aMin, aMax},
    PlotStyle → {Directive[Red, Thick]}, Frame → True, PlotRange → {0, 0.45}];
  eqvsaaGra = Show[Xmgra, Ymgra, Xgra, Ygra, Epilog → thresh,
   FrameLabel → {"Supplemented Amino Acids: a", "Equilibrium cell density"},
   PlotRange → {{Log[0.07], Log[1]}, {0, 0.45}}]
```

Out[710]=

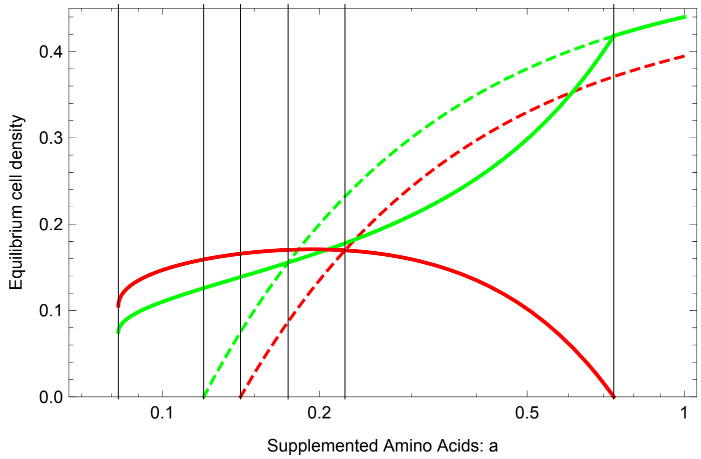

Figure: Eigenvalues and eigenvector of fixed point

In[711]:= $\lambda 6a = \frac{1}{2}\left(tr6 + \sqrt{tr6^2 - 4\,det6}\right)$; $\lambda 6b = \frac{1}{2}\left(tr6 - \sqrt{tr6^2 - 4\,det6}\right)$;

```
In[712]:= spvsaaGra = LogLinearPlot[{λ6a, λ6b}, {a, .08, .8},
   Axes → True, Frame → True, Epilog → thresh, AxesOrigin → {0, 0},
   PlotStyle → {Directive[Magenta], Directive[Black]}, AspectRatio → 1 / 3]
```

Out[712]=

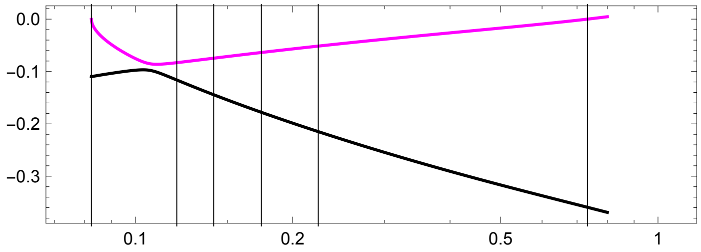


```
In[713]:= boxesAA = {.085, .1066, .12, .3, .6};

In[714]:= figs = Line[{{Log[#], -.1}, {Log[#], 0}}] & /@ boxesAA;
```

```
In[715]:= printBox[aa_] := Module[{xRange = .4, yRange = .4},
   fp6 = {X, Y} /. fp6val /. a → aa;
   g6 = Graphics[{PointSize[.04], Blue, Point[fp6]}];
   J6at1 = J6 /. a → aa;
   {{λeqa, λeqb}, {veqa, veqb}} = J6at1 // Eigensystem;
   veqa2 = veqa / (5 Norm[veqa]); veqb2 = veqb / (5 Norm[veqb]);
   veqaGra = Graphics[{Blue, Arrow[{fp6 - veqa2/2, fp6 + veqa2/2}]}];
   veqbGra = Graphics[{Blue, Arrow[{fp6 - veqb2/2, fp6 + veqb2/2}]}];
   Show[{g6, veqbGra}, FrameLabel → {"X", "Y"},
    PlotRange → {{0, xRange}, {0, yRange}}, Frame → True, AspectRatio → 1]
  ]

In[716]:= printBox /@ boxesAA
```

Out[716]=

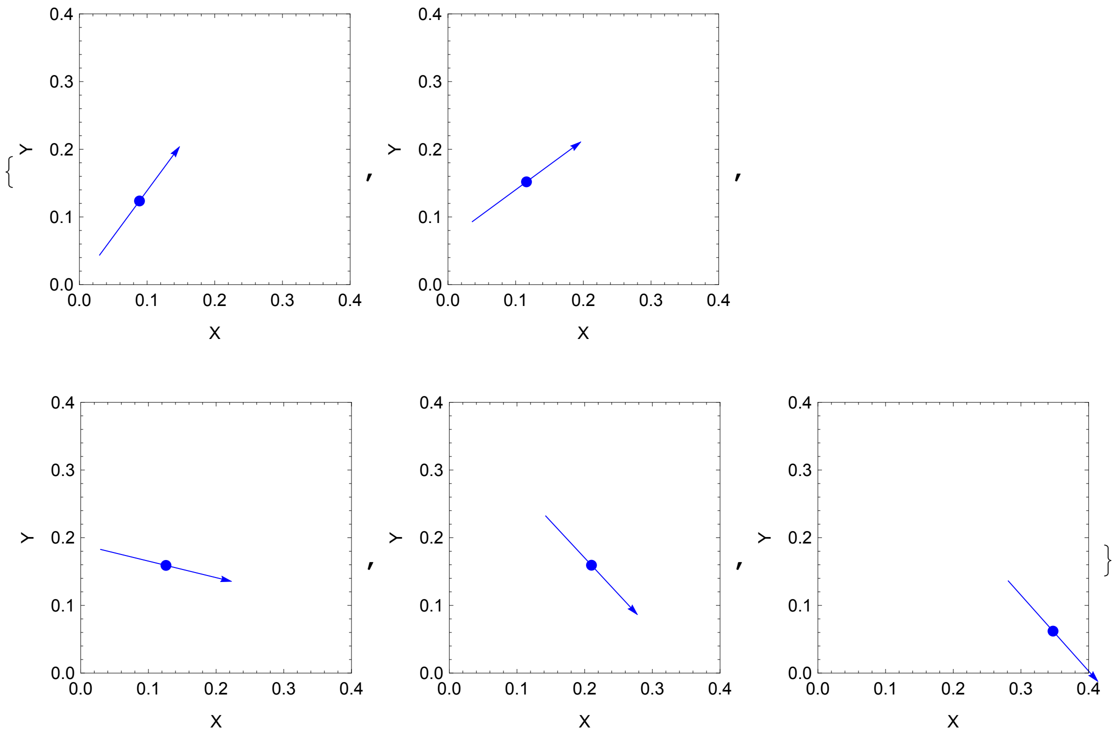

In[717]:=
